# Microbial succession and exploration of higher alcohols-producing core bacteria in northern Huangjiu fermentation

**DOI:** 10.1186/s13568-022-01418-6

**Published:** 2022-06-18

**Authors:** Yi Yan, Leping Sun, Xuan Xing, Huijun Wu, Xin Lu, Wei Zhang, Jialiang Xu, Qing Ren

**Affiliations:** 1 School of Light Industry, Beijing Technology and Business University, Beijing, China; 2 Key Laboratory of Brewing Molecular Engineering of China Light Industry, Beijing, 100048 China; 3 State Key Laboratory for Infectious Disease Prevention and Control, Chinese Center for Disease Control and Prevention, National Institute for Communicable Disease Control and Prevention, Beijing, China; 4 College of Food Science and Technology, Hebei Agricultural University, Baoding, China

**Keywords:** Microbiota, Higher alcohols, Core bacteria, Huangjiu, Fermentation

## Abstract

Higher alcohols (HAs) are abundant compounds that provide important flavors in Huangjiu, but they also cause hangover. Previous studies have shown the production of HAs to be related to yeast, but the correlations between HAs and other microorganisms are rarely reported. In this study, we detected changes in levels of HAs and microbial dynamics during the Huangjiu fermentation process. Relationships were characterized using Pearson’s correlation coefficient. The functional core HA-producing bacteria were selected by bidirectional orthogonal partial least squares (O2PLS). The result showed that 2-methyl-1-propanol, phenethyl alcohol and 3-methyl-1-butanol were the principle HAs present at high levels. *Lactococcus* and *Saccharomyces* were predominant at the genus level of bacteria and fungi, respectively. A total of 684 correlations between HAs and microorganisms were established. Five genera were screened as functional core HA-producing bacteria. Our findings might provide some new inspiration for controlling the content of HAs, enhancing international prestige and market expansion of Huangjiu.

**Supplementary Information:**

The online version contains supplementary material available at 10.1186/s13568-022-01418-6.

## Introduction

Huangjiu is a traditional Chinese alcoholic beverage with unique flavor and high nutritional value (Chen and Xu 2010). At the moment, there have been many varieties of Huangjiu all over China, based on different raw materials, sugar content, production process and geographical regions. Therefore, the volatile compounds and the dynamics of microbial community are quite different. Overall, Huangjiu is produced by three major procedures: selection and soaking of raw materials, followed by alcoholic fermentation, finally comes to post-process treatment (Yang et al. 2020). The usage of *Broomcorn millet* as the basic raw material in northern Huangjiu is the result of its high yields in northern China, while glutinous rice is preferred in southern Huangjiu, which is regionally determined (Han et al. 2019). After continuously simmering in a large saucepan, water evaporation accelerates, also with the enhancement of color. In Shaoxing Huangjiu (a typical representative of southern Huangjiu), the status of *Broomcorn millet* should maintain burning instead of scorching, and this is helpful for coloring in subsequent fermentation process. The color of Huangjiu originates from the natural color of grain, especially *Broomcorn millet* from northern Huangjiu, totally different from the caramel-added yellow rice wine in southern Huangjiu. In addition, the high-temperature gelatinization of the glutinous rice is responsible for the coloring of southern Huangjiu, followed by the steaming process, which might increase the risk of contamination when improper gelatinization. The alcoholic fermentation is very critical in Huangjiu brewing, when the nutrients in raw materials mentioned above are transformed to the aroma and flavors with the help of the microorganisms in the fermentation starters of Qu. In some studies, saccharification is also referred to as primary fermentation while alcoholic fermentation is known as secondary fermentation (Chen et al. 2021). Qu could be classified into yellow Wheat Qu, red Hong Qu and yellow Hong Qu. Wheat Qu is produced in an open environment with nonsterilized, raw wheat as the material. It is a fermentation starter containing various microorganisms and enzyme systems that determine the taste and quality of Huangjiu (Chen and Xu 2013). The microbes during Huangjiu fermentation have been attracting researchers’ interests all along. Thanks to the rapid development of high-throughput sequencing (HTS) and culturomics, large amounts of species have been detected, identified and isolated (Cai et al. 2018; Chen et al. 2020; Liang et al. 2020; Liu et al. 2019, 2020a, 2018, 2020b; Ren et al. 2020). For example, during the fermentation of Shaoxing mechanized Huangjiu, the results showed that bacteria of *Saccharopolyspora*, *Staphylococcus*, *Lactobacillus*, *Streptomyces*, *Actinopolyspora* and *Amycolatopsis*, fungi of *Saccharomyces* and *Aspergillus* are the dominant microorganisms (Liu et al. 2019). In our previous study, *Bacillus*, *Weissella*, *Streptomyces*, *Aeromonas* and *Blautia* were the dominant bacteria in the Huangjiu fermented from corn (Ren et al. 2020). *Saccharomyces cerevisiae* was identified as the predominant yeast during the fermentation of Shaoxing Huangjiu, and the other 10 most abundant bacteria genera were *Bacillus*, *Lactococcus*, *Leuconostoc*, *Staphylococcus*, *Pseudomonas*, *Weissella*, *Thermoactinomyces*, *Saccharopolyspora*, *Enterobacter* and *Lactobacillus* according to Zhang et al.’s and Liu et al.’s work (Liu et al. 2015; Zhang et al. 2012). The analysis of rice wine koji from Hubei province and Sichuan province in China indicated that the main bacterial genera were *Weissella*, *Lactobacillus*, *Lactococcus*, *Bacillus*, *Enterococcus* and *Cronobacter* (Zhao et al. 2020b). Generally, there could be great differences in microbial composition and dynamic changes of different raw materials and fermentation starters.

The aroma of Huangjiu is the key factor determining its sensory quality, which includes massive volatile and non-volatile flavor compounds, also affected by raw materials and fermentation starter mentioned above (Chen et al. 2019; Jiao et al. 2017). The volatile aroma components attribute most to the style and quality of Huangjiu (Jiang et al. 2020a; Sun et al. 2018). Alcohols, esters, acids, aldehydes and various heterocyclic compounds are the main trace components of the volatile compounds (Hu et al. 2019; Yang et al. 2020). The alcohols are composed of ethanol, methanol, n-butanol and n-propanol, providing the sweetness and flavor, also function as the precursors of esters, which consist of ethyl lactate, ethyl acetate and ethyl formate (Wang et al. 2022; Xu et al. 2015). The higher alcohols (HAs) in the complex flavor system serve as important aromatic and organoleptic compounds (Li et al. 2018a). HAs also called fusel oils or fusel alcohols, mainly refer to alcohols possessing more than two carbons, containing isopropyl alcohol, allyl alcohol, 2-methyl-1-propanol, 3-methyl-1-butanol and phenethyl alcohol and much more in fermented wine (Huang et al. 2017; Liu 2015). The current findings suggested that moderate amounts of HAs could enrich the coordinated flavors of wine, but the excess might produce adverse effects on health. HAs contribute to the aromatic complexity of wine at concentrations below 300 mg/L, whereas they are considered to have a negative impact on wine quality at concentrations exceeding 400 mg/L (Cameleyre et al. 2015). There have been studies showing that hangover might be enhanced since the HAs would last longer inside of body when the length of the carbon chains in HAs increased (Greenberg, 1970). Some researchers also found that the effects of hangover differed after consuming vodka (without HAs) and whisky (with HAs) (Bonte and Volck 1978; Cheng 2019; Gou et al. 2016; Murphree et al. 1967; Tian et al. 2017). Previous studies have indicated that HAs are formed by microbial metabolism, either from amino acids in feedstock via the Ehrlich pathway or directly from sugar degradation, also known as Harris pathway (Gonzalez and Morales 2017; Sun and Xiao 2018). α-ketonic acids originated from glycolysis and TCA cycle by glucose could further react with –NH_2_ producing amino acids, along with the formation of HAs with the help of pyruvate decarboxylase and dehydrogenase in Harris pathway (Lilly et al. 2006; Sun et al. 2019). Whereas in Ehrlich pathway, α-ketonic acids are generated from amino acids derived from the protein in *Saccharomyces*. After decarboxylation and reduction, higher alcohols with one carbon less than the original amino acids were accumulated. Valine could produce isobutanol, leucine → isopentanol and phenylalanine → phenylethanol were similar (Lei et al. 2013; Yu et al. 2006). What’s more, during the synthesis process of lipids (including the reaction of acids and glycerol for the formation of esters) and the alcoholic catabolism, fatty alcohols are generated from fatty acids initially (Rizzo et al. 1987). In other words, straight chain alcohols with more than 6 carbon atoms are produced from lipid oxidation products, also known as fatty alcohols. The synthesis of fatty alcohols relies on the reduction of activated forms of fatty acyl-CoA/fatty acyl-ACP catalyzed by a fatty acyl reductase (FAR) or the reduction of free fatty acids catalyzed by an enzyme carboxylic acid reductase (CAR) or the reduction of fatty acids to fatty aldehyde through an acyl-protein intermediate, which has been proved appearing in *Photobacterium phosphoreum* (Krishnan et al. 2020).

The contents and varieties of HAs depend on many factors, ranging from 0.1 to 0.7% in relation to ethanol produced (Pietruszka et al. 2010). Yeasts, especially *Saccharomyces cerevisiae*, are considered to be most responsible for the biosynthesis of HAs among the microbe (Li et al. 2018a; Liu et al. 2021; Pires et al. 2014; Tian et al. 2013). Previously, the study of the formation of HAs mostly focused on the only known HA-producing microbe *Saccharomyces* (Cameleyre et al. 2015; Furdikova et al. 2017; Li et al. 2018b; Ma et al. 2017; Zhang et al. 2015). However, the relationships between other microorganisms and HAs have not been fully understood owing to the complex community structure. The study of bacterial function on HAs generation receives less concern (Tian et al. 2022), and this is the reason why we pay attention to the bacteria related to this process.

With the development of HTS and bioinformatics, statistical and mathematical models have been applied to predict the role of microbiota and the function of the community in the fermentation of food (Cao et al. 2017; Ercolini 2013; Jagadeesan et al. 2019). The Pearson correlation coefficient (r) has been applied to analyze relationships between microbial genera and metabolites and thereby correlate the microbiota with volatile compounds in the fermentation of Baijiu (Wang et al. 2017). In addition, bidirectional orthogonal partial least squares (O2PLS) has been applied to select functional core microbiota by comparing the comprehensive importance of microbiota correlated with certain flavors (Wang et al. 2016).

In this study, we adopted the Pearson correlation coefficient (r), correlation networks and O2PLS to model the relationship between microbial genera and HAs. Five functional core bacteria were correlated with HAs in the process of Huangjiu fermentation. Detection of the changes in HAs and the dynamics of microorganisms of northern Huangjiu, as well as prediction of their relationships and speculation regarding methods to control HAs to increase flavor or reduce hangover, are presented in this study. Our findings might provide some new inspiration for controlling the content of HAs, enhancing international prestige and market expansion of Huangjiu.

## Materials and methods

### Sampling

Semisolid-state fermentation was carried out in Bei-Zong Huangjiu winery’s experimental pit located in Hebei province, China, under a constant temperature of 25 °C. Huangjiu was fermented using *Broomcorn millet* grain as feedstock. The fermentation process followed was according to the conventional fermentation method used in wineries. Wheat Qu and amyloglucosidase were added as starter before fermentation. Wheat Qu was made in an open environment in August 2017. The period of fermentation was 10 days. Three parallel Huangjiu mash samples were collected every two days during the fermentation stages of 0, 2, 4, 6, 8 and 10 days in October 2017. A total of 18 samples were collected. Each sample, approximately 300 g, was divided into 3 parts: 200 g of each sample was used for quality control and HAs testing, approximately 5 g of each sample was used for isolation of strains, and the remainder of each sample was placed in a separate sterile bottle and stored at − 80 °C until DNA extraction. Dry ice was used to maintain low temperature during transportation.

*Broomcorn millet*→Soaking→Steaming→10 days fermentation→Filtering and sterilizing

### Quality control of the fermentation process

The National Standard Method GB/T13662-2018 was employed to determine acidity and alcohol content to evaluate Huangjiu quality, providing reference standards for the detailed operating procedures. A 200 g sample was centrifuged at 4000 r/min for 20 min, and the supernatant was collected. A sample (100 mL) of the supernatant was rotary evaporated, 95 mL of the distillate was collected, and water was added to 100 mL. The alcohol content was measured with an alcohol meter. Acidity of the supernatant during the fermentation process was detected by titration. The dinitro salicylic acid (DNS) method was employed to determine the reducing sugar content with glucose as a reference standard. A 1.0 mL liquid sample was diluted 10 times, and then 1.0 mL of the diluted solution and 1.0 mL of DNS solution were mixed and kept in a boiling water bath for 5 min. The mixture was diluted by distilled water to 10.0 mL when it was cooled to room temperature, and its absorbance at 540 nm was read (Guan et al. 1999).

### Qualitative and semiquantitative analysis of higher alcohols

HAs in each sample were analyzed by headspace solid phase microextraction gas chromatography mass spectrometry (HS-SPME/GC-MS) (Jiang et al. 2020b; Ma et al. 2017; Zhao et al. 2020a). For HS-SPME/GC-MS analysis, each Huangjiu sample (0.5 mL) was placed in a 15-mL solid phase micro-extraction (SPME) glass vial together with 5.44 mL ethanol (15% volume fraction) and 60 μL of the internal standard 2-octanol (8800 µg/L). The 50/30 µm divinylbenzene/carboxen/polydimethylsiloxane (DVB/CAR/PDMS) extraction fiber was inserted into the vial. The sample vials were bathed in 50 °C water and ultrasonic extracted for 45 min. After extraction, fiber was inserted into the injection port of the gas chromatography mass spectrometry (GC–MS) system and thermally desorbed at 250 °C for 5 min.

The analysis was carried out on a Shimadzu-QP2010 Plus-GCMS. Each enriched compound was analyzed by DB-wax column (30 m × 0.25 mm i.d., 0.25 µm film thickness). The operating condition for GC was as follows: high-purity helium (purity > 99.999%) as the carrier gas without split flow, flow rate at 1.0 mL/min, and temperature of injection port at 250 °C. The column oven temperature program began with 40 °C for 3 min, increased at a rate of 6 °C/min up to 100 °C, and then increased at a rate of 10 °C/min up to 230 °C for 7 min. The temperature of the injector and detector was 250 °C, the temperature of the ion source was 230 °C, the ionization mode was electronic ionization (EI), the EI emission current was 50 μA, and the EI ion energy was 70 eV. The chromatograms were recorded by monitoring the total ion currents in the range of 33–400 mass.

HA content in Huangjiu was calculated by substituting peak area of HA and internal standard detected by GC–MS into Eq. (1).

1 $$ {\text{C}} = 12{\text{A}}_{{\text{c}}} /{\text{A}}_{{{\text{is}}}} \times {\text{C}}_{{{\text{is}}}} $$

In the equation: C: content of HA in Huangjiu, µg/L; C_is_: content of the internal standard in sample, µg/L; A_c_: peak area of HA in Huangjiu; A_is_: peak area of the internal standard.

### DNA extraction, PCR amplification and Illumina MiSeq sequencing

Microbial DNA was extracted from the samples by the E.Z.N.A.^®^ soil DNA kit (OMEGA Bio-tek, America). The quality of genomic DNA quality was detected by 1% agarose gel electrophoresis. The V3-V4 hypervariable regions of the bacterial 16S rRNA gene were amplified by thermocycler polymerase chain reaction (PCR). The thermocycler PCR system process was set at: 95 °C for 5 min; 25 cycles at 95 °C for 30 s, 55 °C for 30 s and 72 °C for 40 s; followed by extension at 72 °C for 10 min. Primers were 338 F (5′-ACTCCTACGGGAGGCAGCAG-3′) and 806 R (5′-GGACTACHVGGGTWTCTAAT-3′). The internal transcribed spacer ITS1 region of fungi was amplified by PCR with a system process set at: 95 °C for 2 min; 30 cycles at 95 °C for 30 s, 61 °C for 30 s, and 72 °C for 45 s; and extension at 72 °C for 10 min. Primers were ITS1 (5′-AxxxCTTGGTCATTTAGAGGAAGTAA-3′) and ITS2 (5′-BGCTGCGTTCTTCATCGATGC-3′) (Buee et al. 2009). The final DNA concentration and purity were determined using a NanoDrop 2000 UV–vis spectrophotometer. The amplicons were then pooled in equimolar concentrations into a single tube in preparation for paired-end sequencing (2 × 300 bp) (Ren et al. 2015) on an Illumina MiSeq platform according to standard protocols provided by Majorbio Bio-Pharm Technology Co. Ltd. (Shanghai, China). The fungal and bacterial sequence data reported in this study have been archived in the Sequence Read Archive (SRA) database with the accession number SRP303298, SRP303296, respectively.

### Processing of sequencing data

Quality filtration by Trimmomatic eliminated unqualified samples, and reads were merged by fast length adjustment of short reads (FLASH) under the following principle: first, the reads were truncated at any site receiving an average quality score < 20, setting a 50-bp sliding window. Second, according to the overlap relationship between paired-end DNA sequencing (PE) reads, the pairs of reads were merged into a sequence, with a minimum overlap length was 10 bp. Third, the maximum mismatch ratio allowed for the overlap area of the paired-end sequence was 0.2, and the reads containing ambiguity bases were deleted. Finally, primers were removed by allowing two nucleotide mismatches (Magoc and Salzberg 2011).

Operational taxonomic units (OTUs) clustering was performed on all sequences with 97% identity cutoff by UPARSE software (version 7.1). Chimeric sequences were identified and deleted by UCHIME. The taxonomy of the 16S rRNA gene sequence was analyzed by the ribosomal database project (RDP) Classifier algorithm against the Silva (SSU123) 16S rRNA database with 70% confidence threshold (Cole et al. 2009; Gurevich et al. 2013; Klindworth et al. 2013). The taxonomy of each ITS gene sequence was analyzed by Unite (Release 6.0) (Koljalg et al. 2013). Alpha rarefaction was performed in QIIME (version 1.7.0) using Chao1 to estimate species abundance (Caporaso et al. 2010). Species richness was estimated by the number of unique operational taxonomic units (OTUs). The Simpson index of OTU was calculated. The greater the value of the Simpson index, the lower the diversity of the community (Hill et al. 2003). Network analysis was used for correlating species abundance information between different samples to obtain the coexistence relationship and the interaction of species in the same environment.

### Prediction of correlation between microorganisms and HAs

Correlations between the microbial genera and HAs were established by Pearson correlation coefficient (r) to predict the relationship of the microbiota with the HA composition. The P value was adjusted by false discovery rate (FDR) using the Benjamini–Hochberg method. The cutoff for P and for the adjusted P value was set at 0.05. The correlation network was constructed by all significant associations and displayed using R programming language.

### Exploring HA-producing core bacteria in northern Huangjiu

To further integrate the microorganisms and HA data, O2PLS analysis was performed. Datasets were preprocessed as described by Bylesjö et al. (Bylesjo et al. 2007). In this method, both datasets were mean-centered by feature element, and the HA data were scaled to unit variance for each resolved peak. In addition, both datasets were scaled to an equal total sum of squares of 1. Finally, the O2PLS model was built by the OmicsPLS package of R34. A permutation test was performed to establish a threshold for identifying the most influential variables. The normalization of matrix involves subtracting the mean of each column and then dividing by the standard deviation. Datasets were reshuffled 1000 times and the O2PLS model was established for each permutation. Significance level (α) was set as 0.05 for the two datasets. Latent variables were the lower and upper α/2 quantiles of the loading values (Rodriguez et al. 2018). Further statistical and graphical analyses were performed in Excel and R software.

## Results

### Quality control of the fermentation process

**Fig. 1:**
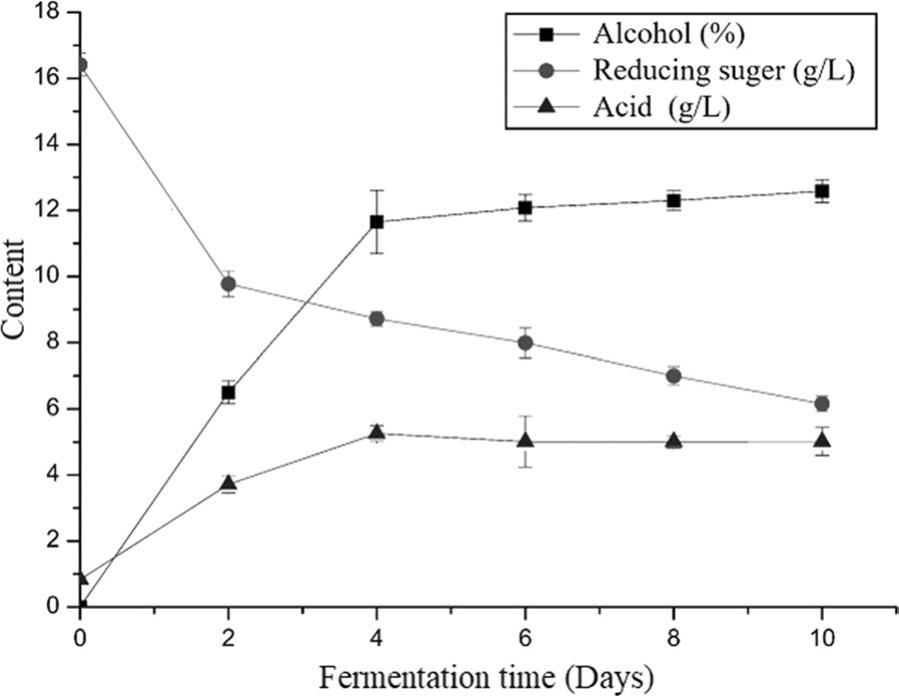
The changes in alcohol, reducing sugar and acid during fermentation of northern Huangjiu. Square indicates the content of alcohol. Circle indicates the content of reducing sugar. Triangle indicates the content of acid

Experimental data for quality control are shown in Fig. 1. The data indicated that content of alcohol and acid increased with the fermentation stage, and alcohol content increased most during the first two days of fermentation. During the fermentation period of the 2nd to the 4th days, alcohol content increased significantly in the fermenter. From the 4th to the 10th day of fermentation, alcohol content increased slightly and stabilized. During the fermentation process, reducing sugar content decreased with fermentation time. In the first two days of fermentation, reducing sugar content dropped sharply and coincided with a trend of rising alcohol content. From the 2nd to the 10th day, the content of reducing sugar gradually decreased and tended to stabilize. The acid content first increased and then tended to plateau. Finally, the acid content was approximately 5 g/L, within the standard of corruption. All of the above indicators meet the national standard GB/T-13662-2008 for Huangjiu. Detailed data are attached in Additional file 1: Table S1.

### Qualitative and semiquantitative analysis of higher alcohols

A total of 23 kinds of HAs were detected in the samples over 10 days (Table 1). HAs with high content (> 1.000 mg/L detected at one time) included 2-methyl-1-propanol, phenethyl alcohol, 1-triacontanol, 3-methyl-1-butanol, 1-hexacosanol, 2-o-decyl-threitol, 2-ethyl-2-methyl-tridecanol, 2-furanmethanol, 2-phenoxy-ethanol and 2-(2-butoxyethoxy)-ethanol. In addition, 1-dodecanol and 1-hexadecanol were steady. The total concentration of HA exhibited an upward trend and reached the maximum of 31.39 mg/L on the 10th day. However, only eleven kinds of HAs were detected on the last day of fermentation.

**Table 1:** The changes of HA during the fermentation of northern Huangjiu

HA (μg/L)	Day 0	Day 2	Day 4	Day 6	Day 8	Day 10
Phenylethyl Alcohol	3622.04 ± 41.20	1044.49 ± 27.29	1514.35 ± 58.50	2186.36 ± 67.92	3636.99 ± 65.38	3935.78 ± 26.14
2-methyl-1-Propanol	ND	33.79 ± 1.66	57.62 ± 2.02	273.63 ± 10.84	489.67 ± 5.50	16,280.09 ± 759.33
3-methyl-1-Butanol	ND	570.81 ± 5.09	816.92 ± 11.53	3896.08 ± 135.25	4922.36 ± 22.90	3830.97 ± 93.43
2-phenoxy-Ethanol	1360.17 ± 29.66	251.37 ± 8.64	66.17 ± 1.44	254.19 ± 7.46	439.94 ± 12.84	924.99 ± 14.49
1-Triacontanol	ND	175.23 ± 4.79	40.41 ± 0.60	10,605.38 ± 703.62	3509.15 ± 128.55	1040.19 ± 70.22
1-Hexacosanol	ND	343.02 ± 19.80	100.64 ± 0.71	6555.62 ± 217.16	2045.77 ± 32.04	1250.21 ± 64.71
1-Dodecanol	ND	96.87 ± 1.95	19.54 ± 0.92	444.37 ± 31.65	873.75 ± 24.19	391.80 ± 4.39
1-Hexadecanol	ND	19.48 ± 0.40	80.71 ± 2.16	518.09 ± 23.46	925.01 ± 8.90	305.18 ± 3.88
2-O-decyl-Threitol	3669.00 ± 34.11	46.34 ± 1.99	128.94 ± 3.39	434.45 ± 15.03	731.42 ± 5.80	634.47 ± 20.50
2-ethyl-2-methyl-Tridecanol	ND	12.68 ± 1.42	13.28 ± 0.12	1023.48 ± 52.55	2513.16 ± 26.41	1653.45 ± 38.04
2-(2-butoxyethoxy)-Ethanol	ND	93.29 ± 1.77	95.33 ± 2.56	493.28 ± 6.34	772.57 ± 20.62	1143.66 ± 45.92
6-Methyl-1-octanol	ND	ND	ND	517.20 ± 16.00	ND	ND
2-Furanmethanol	3550.90 ± 42.75	118.16 ± 2.40	70.06 ± 1.16	ND	ND	ND
Octaethylene glycol	542.35 ± 26.68	57.55 ± 2.43	25.39 ± 2.39	ND	ND	ND
9,12-Octadecadien-1-ol	ND	464.43 ± 13.11	186.01 ± 7.77	ND	ND	ND
2-ethyl-1-Hexanol	ND	54.63 ± 3.49	58.92 ± 0.18	ND	ND	ND
2-Isopropyl-5-methyl-1-heptanol	ND	27.80 ± 1.29	60.70 ± 0.45	ND	ND	ND
3,7-dimethyl-1,6-Octadien-3-ol	ND	46.97 ± 1.39	16.98 ± 0.73	ND	ND	ND
3-(methylthio)-1-Propanol	ND	47.58 ± 0.49	16.58 ± 0.32	ND	ND	ND
1-Heptadecanol	ND	33.45 ± 0.68	24.83 ± 0.62	ND	ND	ND
1-Undecanol	ND	ND	28.77 ± 0.65	ND	ND	ND
5-methyl-2-(1-methylethenyl)-4-Hexen-1-ol	ND	19.47 ± 0.19	ND	ND	ND	ND
4-methyl-1-Heptanol	ND	13.29 ± 0.22	ND	ND	ND	ND

Every value was expressed as means standard error (n = 3)

*ND* no detection

### Fungal diversity during the fermentation process in northern Huangjiu

**Fig. 2:**
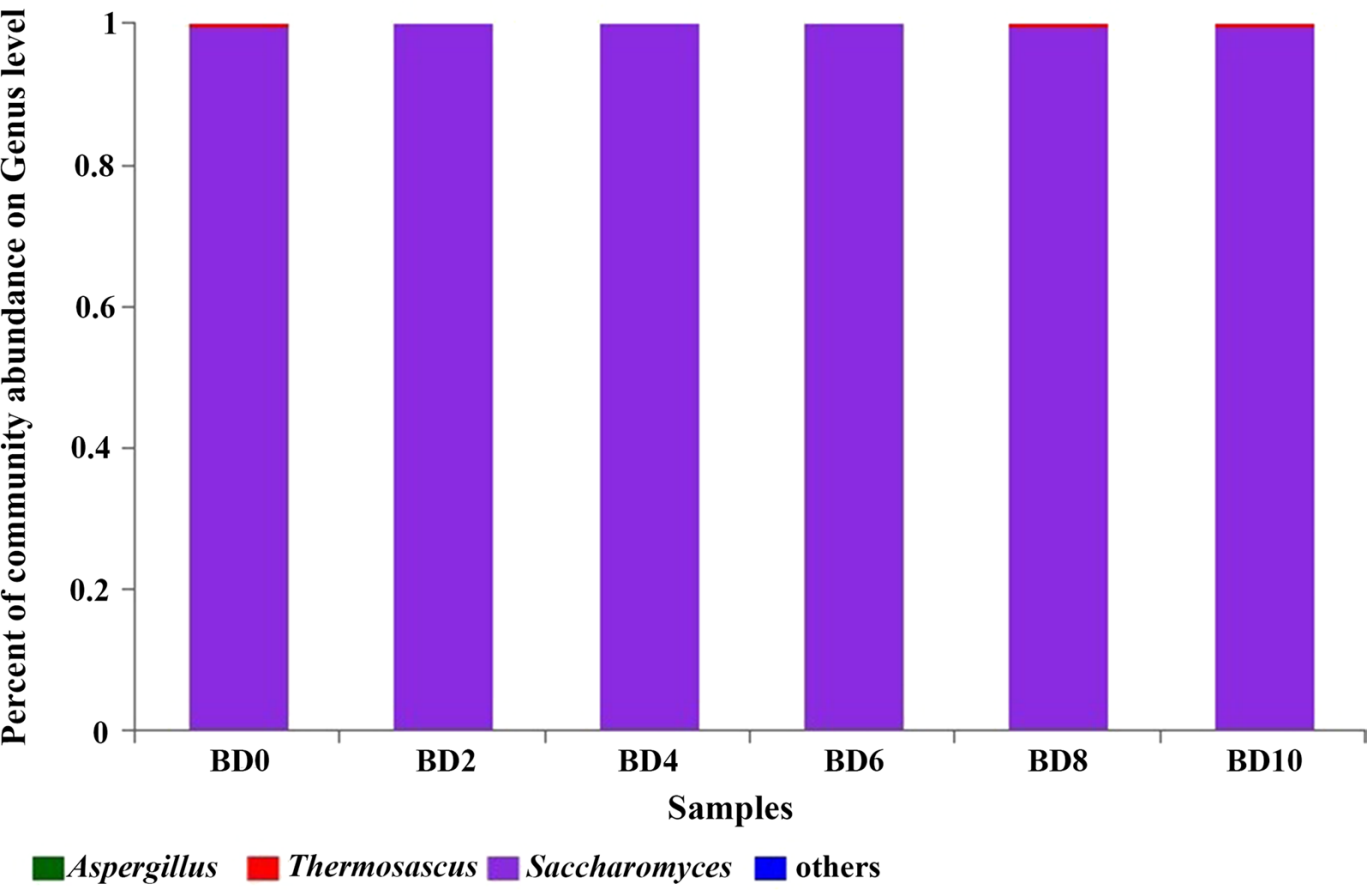
Fungal diversity during the fermentation process in northern Huangjiu at genus level. BD means “Beizong Huangjiu fermentation Day”

The high-throughput sequencing results showed that 678,552 sequencing fragments in 18 Huangjiu samples met the quality control requirements, and 7 OTUs were obtained after clustering (Additional file 1: Table S2). According to the results from comparison with the database, there were 2 phyla, 4 classes, 4 orders, 5 families and 5 genera of fungi at each classification level. The average of three parallel samples was shown in Fig. 2. *Saccharomyces* occupied an absolute predominance in all samples at the genus level. A small amount of *Thermoascus* and *Aspergillus* were also found in the samples (Fig. 2).

To analyze the fungal community diversity of the samples, alpha-diversity index analysis was performed. The result of alpha-diversity index analysis showed that the Simpson index value was same at different fermentation stages (Additional file 1: Figure S1). There was no difference in the fungal community diversity of the samples.

### Bacterial diversity during the fermentation process in northern Huangjiu

**Fig. 3:**
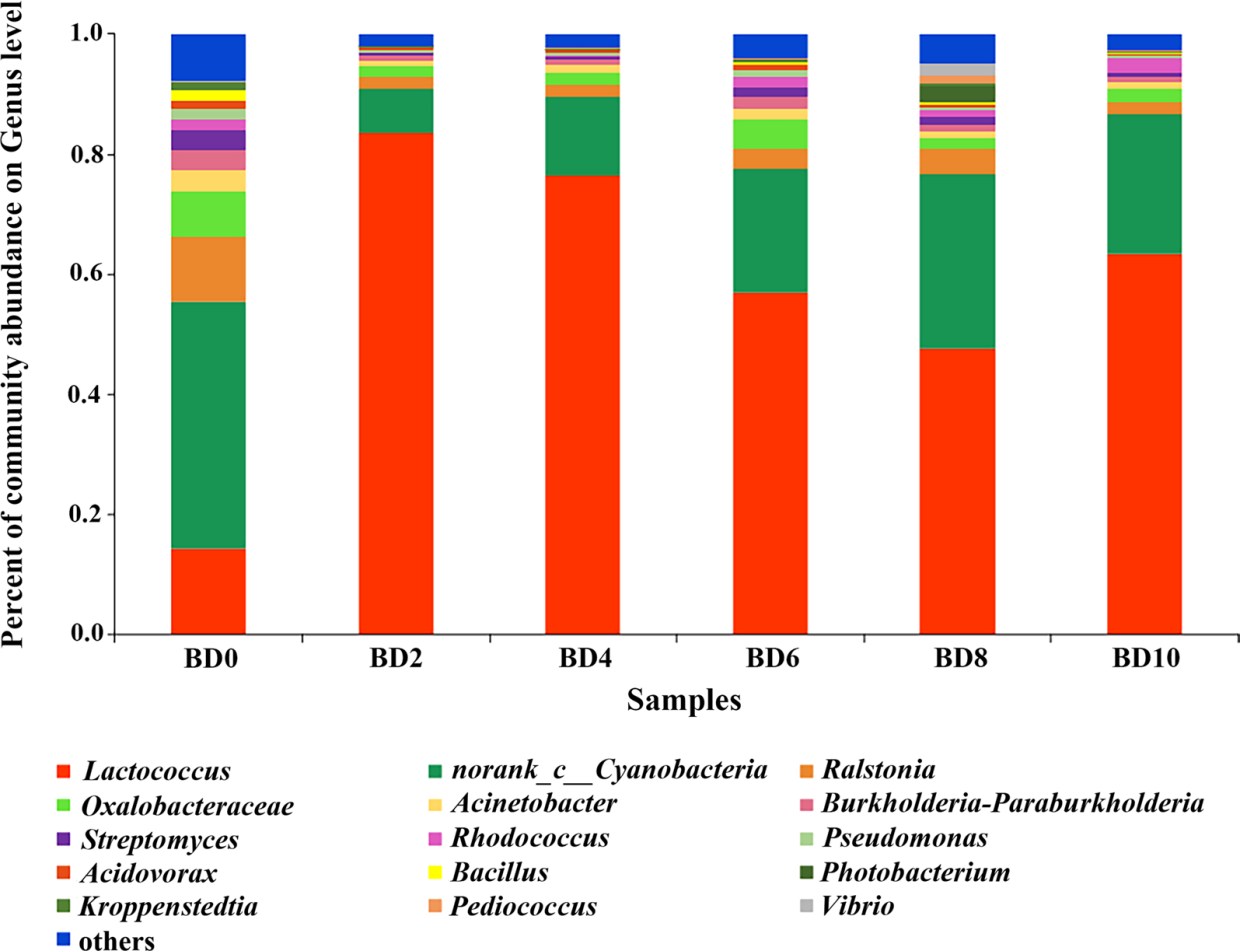
Bacterial diversity during the fermentation process in northern Huangjiu at genus level. BD means “Beizong Huangjiu fermentation Day”

The high-throughput sequencing results showed that 817,386 sequencing fragments in 18 Huangjiu samples met the quality control requirements, and 580 OTUs were obtained after clustering (Additional file 1: Table S3). The results from comparison with the database indicated that there were 27 phyla, 49 classes, 103 orders, 178 families and 335 genera of bacteria at each classification level. The average of three parallel samples was shown in Fig. 3. At the genus level, *Lactococcus* occupied predominance in samples (Fig. 3). Bacterial abundances showed obvious diversity in different fermentation stages.

Alpha-diversity index analysis was used to analyze bacterial community diversity. The result of alpha-diversity index analysis showed that the Simpson index value was clearly different in different fermentation stages (Additional file 1: Figure S2). The diversity of the bacterial community decreased significantly from the 0th to the 2nd day and rose gradually from the 2nd to the 8th day. Samples of the 2nd and the 4th days showed the highest values of the Simpson index, indicating that bacterial community diversity in these two samples were minimal. At the beginning of fermentation, bacterial community diversity was greatest.

Network analysis was performed to obtain the coexistence relationship and the interaction of species. Network analysis of bacterial dynamics during Huangjiu fermentation at the genus level showed that *Lactococcus*, which was the most frequent bacterial genus, was negatively correlated with most bacterial genera. *Escherichia-Shigella* only established correlation with *Lactococcus*. *Spongiimonas*, *Vibrio*, *Photobacterium* and *Pediococcus* built positive correlations with each other and were not associated with other bacteria.

### Correlation between microorganisms and HAs

**Fig. 4:**
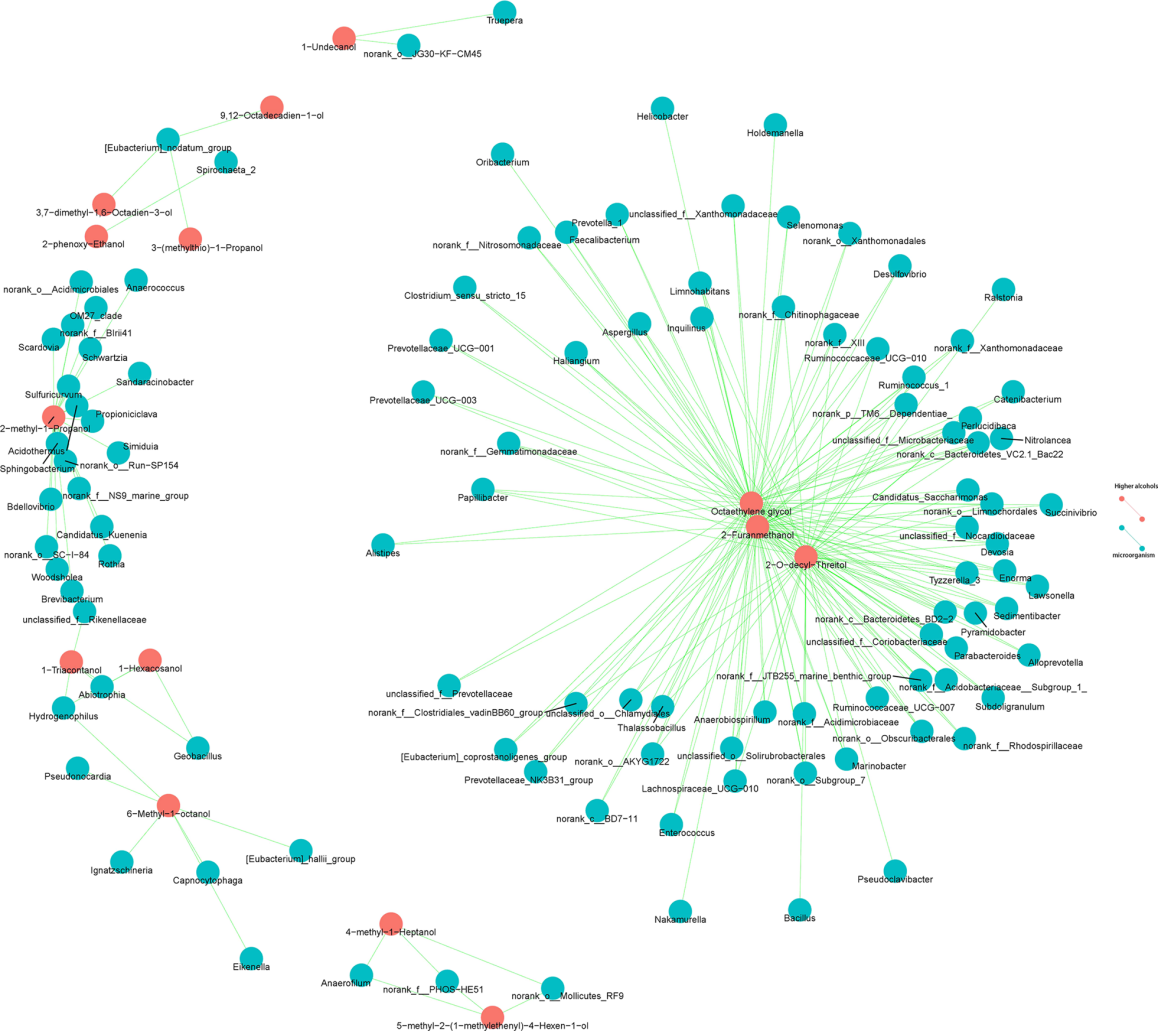
Correlation between microorganisms and higher alcohols. The green line indicates correlation was built between HAs and microorganisms; the blue pie indicates microbiota composition; the red pie indicates HA composition

The correlations between the microbial genera and HAs were tested by Pearson correlation coefficient (r), r > 0.95 was considered as a robust correlation (Wang et al. 2017). The results showed that HAs exhibited 684 correlations with 271 genera of microorganisms. Microorganisms with high abundance (≥ 1%) were correlated with octaethylene glycol, 2-o-decyl-threitol, 2-furanmethanol, 2-ethyl-1-hexanol, 1-hexadecanol, 1-heptadecanol and 1-dodecanol. Additionally, 2-methyl-1-propanol, 3-methyl-1-butanol, 3-(methylthio)-1-propanol, phenethyl alcohol and 2-phenoxy-ethanol, which are always found in Huangjiu, were correlated with 33 genera, 6 genera, 7 genera, 12 genera and 19 genera of microorganisms, respectively. Additionally, 1-triacontanol, 1-hexacosanol, 2-o-decyl-threitol and 2-ethyl-2-methyl-tridecanol with higher content were correlated with 22 genera, 21 genera, 121 genera and 9 genera of microorganisms, respectively. The P values were adjusted by FDR for precise association. (Fig. 4 and Additional file 1: Table S4).

### Exploring HA-producing core bacteria in northern Huangjiu

**Fig. 5:**
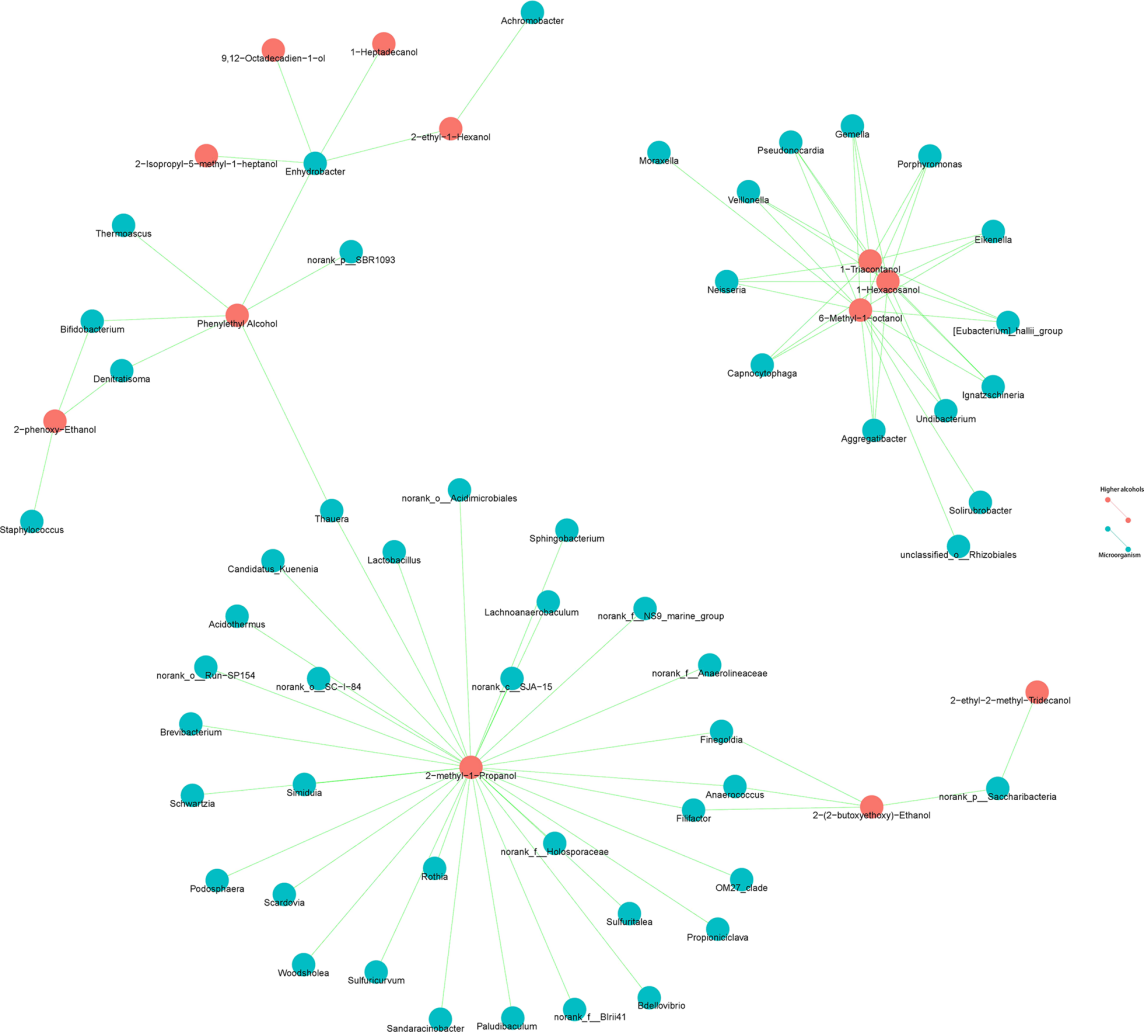
Correlation between HA-producing core bacteria and higher alcohols. The green line indicates correlation was built between HAs and microorganisms; the blue pie indicates micro-biota composition; the red pie indicates HA composition

An O2PLS model was used to study the HA-producing functional core microbiota in Huangjiu. A total of 59 genera were selected as significant HA-producing microbiota. Among them, *Lactobacillus* and *Weissella* etc. had upper quantiles. *Thermoascus*, *Podosphaera paludibaculum* and others had lower quantiles (Additional file 1: Table S5). The correlations between the significant microbiota and HAs were built; 53 genera of significant microbiota built 86 correlations with 12 HAs (p ≤ 0.05). Among them, 2-methyl-1-propanol established the most correlations and was related to 31 genera of microorganisms (Additional file 1: Table S6). For a bacterial genus to be considered functional core HA-producing bacteria in Huangjiu, two criteria needed to be met: (i) its abundance had to be high (top 20%), and (ii) it had to be correlated with stable HAs (was detected ≥ 5 times). Based on these, five genera (*Lactobacillus*, *Neisseria*, *Staphylococcus*, *Thauera* and *Bifidobacterium*) were selected as functional core HA-producing bacteria in Huangjiu. They were related to the production of 2-methyl-1-propanol, 1-hexacosanol, 1-triacontanol, 2-phenoxyethanol and phenethyl alcohol (Fig. 5).

## Discussion

Previous studies have shown that HAs in fermentation processes are mainly produced by yeast metabolism, including the degradative metabolic pathway (Ehrlich pathway) and the anabolic metabolic pathway (Harris pathway) (Cheng et al. 2011). In the Ehrlich pathway, amino acids form HAs by the catalysis of transaminase, ketoacid decarboxylase and aldehyde dehydrogenase. For example, the production of phenethyl alcohol, 2-methyl-1-propanol and 3-methyl-1-butanol are related to L-phenylalanine, valine and leucine, respectively (Hazelwood et al. 2008; Lambrechts and Pretorius 2000). Although amino acid contribution to production of HAs was clear, the input from central carbon metabolism was by no means negligible. In the Harris pathway, glucose forms pyruvate by glycolysis. Pyruvic acid enters the amino acid biosynthesis pathway under the catalysis of acetohydroxy acid synthase and forms an α-keto acid intermediate in the final stage of anabolism. Finally, HAs are produced by enzymatic catalysis.

Phenethyl alcohol is one of the important HAs in Huangjiu. It is an aromatic alcohol with rose-like fragrance which is naturally found in essential oils of many flowers and plants (Chen et al. 2017). In addition, phenethyl alcohol can inhibit some gram-negative bacteria and fungi (Fraud et al. 2003). In this study, *Lactococcus* was inhibited in the middle and last stages of fermentation. This may be due to the increase in phenethyl alcohol content from the 2nd to the 10th day. The main metabolic pathways producing phenethyl alcohol are phenylalanine metabolism and biosynthesis of plant secondary metabolites. In the Ehrlich pathway, phenethyl alcohol is synthesized from L-phenylalanine by transamination to phenylpyruvate, followed by decarboxylation to phenylacetaldehyde and reduction to phenethyl alcohol (Etschmann et al. 2002). Previously, various microorganisms including *Cladosporium cladosporioides*, *Kluyveromyces lactis*, *Saccharomyces cerevisiae*, *Hansenula anomala* and *Kluyveromyces marxianus* have been reported to be capable of producing phenethyl alcohol (Etschmann et al. 2003; Hui 2006). Comparing the microorganisms that were associated with phenethyl alcohol in this study with the Kyoto Encyclopedia of Genes and Genomes (KEGG) database, four microorganisms involved in metabolism of phenethyl alcohol were consistent with those in the KEGG database. They are *Bifidobacterium*, *Bradyrhizobium*, *Lysinibacillus*, *Spirochaeta_2* and *Thauera* (Arai et al. 1999; Diaz et al. 1998; Ferrandez et al. 1997; Hwang et al. 2009; Teufel et al. 2010). In this study, *Denittrasoma*, *Enhydrobacter* and *Thermoascus* were positively correlated with phenylethyl alcohol. These genera are potential producers of phenylethyl alcohol. In addition, *Bifidobacterium* and *Thauera*, which were the HA-producing functional core bacteria, showed positive correlations with phenethyl alcohol. Therefore, they may make the greatest contribution to the production of phenethyl alcohol other than yeast.

2-methyl-1-propanol (isobutyl alcohol) is an important raw material for artificial musk and essential oils (Bauer et al. 2008). It can be biosynthesized from the 2-ketoisovalerate (KIV) biosynthetic pathway and the Ehrlich pathway (Atsumi et al. 2008; Li et al. 2012a, 2012b). Dickinson showed that a single isozyme of pyruvate decarboxylase can form isobutyl alcohol from valine (Dickinson et al. 1998). *Clostridium* is natural producer of 2-methyl-1-propanol, and it can produce quantities of 2-methyl-1-propanol under suitable fermentation conditions (Peralta-Yahya et al. 2012). In this study, the metabolism of 2-methyl-1-propanol was related to 33 genera, such as *Acidothermus*. *Lactobacillus* and *Thauera*, which are HA-producing functional core bacteria and showed negative and positive correlations with 2-methyl-1-propanol, respectively. Therefore, *Thauera* was predicted to contribute to the production of 2-methyl-1-propanol. *Lactobacillus* may have inhibited or decomposed 2-methyl-1-propanol.

3-methyl-1-butanol (isoamyl alcohol) is one of the compounds with the highest potential sensory impact in wines (Gomez-Miguez et al. 2007). It also showed a high proportion in this study. Therefore, controlling the yield of isoamyl alcohol was significant for controlling the content of HA in the fermentation of Huangjiu. Leucine can be metabolized via the Ehrlich pathway to form isoamyl alcohol in *Saccharomyces* (Atsumi et al. 2008). However, the content of isoamyl alcohol did not strictly increase with increasing abundance of yeast (Sun 2012). The production of isoamyl alcohol was the result of a variety of microbial and environmental interactions. In this study, isoamyl alcohol established correlation with 6 genera. It was positively associated with *Cloacibacterium* and negatively associated with *Anaerrotruncus*, *Eubacterium eligens*, *Odoribacter*, *Lachnospiraceae_NK4A136_group* and *norank_f_Erysipelotrichaceae*. However, because of the lack of significance and low abundance, 3-methyl-1-butanol may be most closely related to *Saccharomyces*.

2-phenoxyethanol is an ingredient used for many fragrance products. It is a colorless liquid with a mildly rosy aroma (Scognamiglio et al. 2012). In this study, 2-phenoxyethanol was associated with 19 genera. It was negatively correlated with *Saccharomyces* and *Lactococcus* and positively correlated with *Staphylococcus*, *Denitratisoma* and *Bifidobacterium*, which were significant microorganisms. Furthermore, *Staphylococcus* and *Bifidobacterium* were screened as HA-producing functional core bacteria. Therefore, *Staphylococcus* and *Bifidobacterium* may make a high contribution to the production of 2-phenoxyethanol.

Among the 23 HAs, there were 6 kinds of alkanols, including 1-triacontanol, 1-hexacosanol, 1-dodecanol, 1-hexadecanol, 1-heptadecanol and 1-undecanol. 1-hexacosanol, 1-octacosanol and 1-triacontanol have been studied and discussed extensively. 1-hexacosanol (cerylalcohol) shows anti-fatigue, anti-tumor, immunity, anti-cholesterol, and neuroprotective action and provides nutrition for nerves, greatly reducing the degradation of cholinergic neurons. 1-triacontanol (myricylalcohol) is mostly present in the form of esters in a variety of plants. It is a natural plant growth regulator, which has special regulatory effects on the growth of plants and no toxic effects on humans or animals (Duan et al. 2005). However, alkanols play a negative role in wine’s aroma quality (de-la-Fuente-Blanco et al. 2016). Therefore, it is necessary to control the microorganisms that produce alkanols during the fermentation process. There are mainly three pathways for alkanol synthesis in microorganisms: fatty acyl-ACP as substrate, free fatty acids as substrates and fatty acyl-CoA as substrate (Cao et al. 2015; Liu et al., 2013, 2014; Lu et al. 2016). However, the above-referenced studies focused on constructing genetically engineered microorganisms. However, the correlation between alkanols and wild-type strains has rarely been reported. In this study, six alkanols were associated with 83 genera. 1-triacontanol and 1-hexacosanol showed positive correlations with *Neisseria,* a functional core bacterial genus; therefore, *Neisseria* may have contributed substantially to their production.

The type of HA varies little among different Huangjiu wines. Liu et al. (2019) found that β-phenylethanol, isoamyl alcohol and isobutanol were the most abundant HAs in Shaoxing mechanized Huangjiu. It is the same as this study. HAs are important flavor compounds; their sources are mainly studied through metabolic pathway analysis and mathematical model prediction. *Saccharomyces* and non-*Saccharomyces* yeasts (*Pichia mississippiensis* and *Wickerhamomyces anomalu*s) have generally been considered to be the main producers of HAs, especially during the prefermentation stage of Huangjiu, based on O2PLS-based correlation analysis (Huang et al. 2018). The results of a flavor metabolic network, which was constructed using the KEGG database and information from the literature, indicated that *Streptomyces*, *Staphylococcus*, *Lactobacillus*, *Aspergillus* and *Choanephora* are potential producers of HAs (Liu et al. 2019). In this study, *Lactobacillus*, *Neisseria*, *Staphylococcus*, *Thauera* and *Bifidobacterium* were selected as functional core bacteria for producing HAs in Huangjiu. *Lactobacillus* and *Staphylococcus* were designated as HA producers under both methods. However, *Neisseria*, *Thauera* and *Bifidobacterium* were bacterial genera without high relative abundance in Shaoxing Huangjiu. Therefore, the differences in HA-producing core bacteria under different analytic methods may be caused by the discrepancy in microbial diversity among different samples.

In this study, a total of 23 HAs were detected. 2-methyl-1-propanol, phenethyl alcohol and 3-methyl-1-butanol were the principle HAs in northern Huangjiu. *Lactococcus* and *Saccharomyces* predominated at the genus level of bacteria and fungi, respectively. 684 correlations between HAs and microorganism were built. In addition, *Lactobacillus*, *Neisseria*, *Staphylococcus*, *Thauera* and *Bifidobacterium* were screened as functional core bacteria for producing HAs in Huangjiu. They were related to the production of 2-methyl-1-propanol, phenethyl alcohol, 2-phenoxy-ethanol, 1-triacontanol and 1-hexacosanol using the Pearson correlation coefficient.

The production of HAs during the fermentation of Huangjiu is the result of a variety of microbial and environmental interactions. However, in addition to yeast, the role of other microorganisms in the production of HAs is not clear. The prediction and verification of the correlations between HAs and microorganisms have great significance for controlling HA content, the selection of fermentation strains and the quality control of Huangjiu. Through the selection or elimination of strains, the types and content of HAs can be controlled, thereby achieving the effect of increasing flavor or reducing the sense of a hangover.

## Supplementary Information

**Additional file 1: Figure S1.** The fungal community diversity of the samples using alpha-diversity index. **Figure S2.** The bacterial community diversity of the samples using alpha-diversity index. **Table S1.** The changes of alcohol, reducing sugar and acid during fermentation in northern Huangjiu. **Table S2.** The high-throughput sequencing of fungal diversity during the fermentation process in northern Huangjiu. **Table S3.** The high-throughput sequencing of bacterial diversity during the fermentation process in northern Huangjiu. **Table S4.** Correlation between HA and microorganisms on genus level. **Table S5.** Significant microbiota for HA-producing. **Table S6.** Correlation between HA and significant microbiota on genus level.

## Data Availability

All data generated or analyzed in this study have been included in this manuscript and additional file.
