# Supplementary material for: Microbial succession and exploration of higher alcohols-producing core bacteria in northern Huangjiu fermentation
Source: AMB Express. 2022 Jun 18;12:79. doi: 10.1186/s13568-022-01418-6 (PMC9206695; doi:10.1186/s13568-022-01418-6)
Supplement: Supplementary file 1 — Additional file 1: Figure S1. The fungal community diversity of the samples using alpha-diversity index. Figure S2. The bacterial community diversity of the samples using alpha-diversity index. Table S1. The changes of alcohol, reducing sugar and acid during fermentation in northern Huangjiu. Table S2. The high-throughput sequencing of fungal diversity during the fermentation process in northern Huangjiu. Table S3. The high-throughput sequencing of bacterial diversity during the fermentation process in northern Huangjiu. Table S4. Correlation between HA and microorganisms on genus level. Table S5. Significant microbiota for HA-producing. Table S6. Correlation between HA and significant microbiota on genus level. [file 13568_2022_1418_MOESM1_ESM.pdf]

Microbial Succession and Higher Alcohols Production in Northern Huangjiu Fermentation

Yi Yan[1,2], Leping Sun[1,2], Xuan Xing[1,2], Huijun Wu[1,2], Xin Lu[3], Wei Zhang[4], Jialiang Xu[1,2*], Qing Ren[1,2*]

[1]School of Light Industry, Beijing Technology and Business University, Beijing, China

[2]Key Laboratory of Brewing Molecular Engineering of China Light Industry, Beijing 100048, China

[3]State Key Laboratory for Infectious Disease Prevention and Control, National Institute for Communicable Disease Control and Prevention, Chinese Center for Disease Control and Prevention, Beijing, China

[4]College of Food Science and Technology, Hebei Agricultural University, Baoding, China

*** Correspondence:**

Jialiang Xu

xujialiang@btbu.edu.cn

Qing Ren
renqing@th.btbu.edu.cn

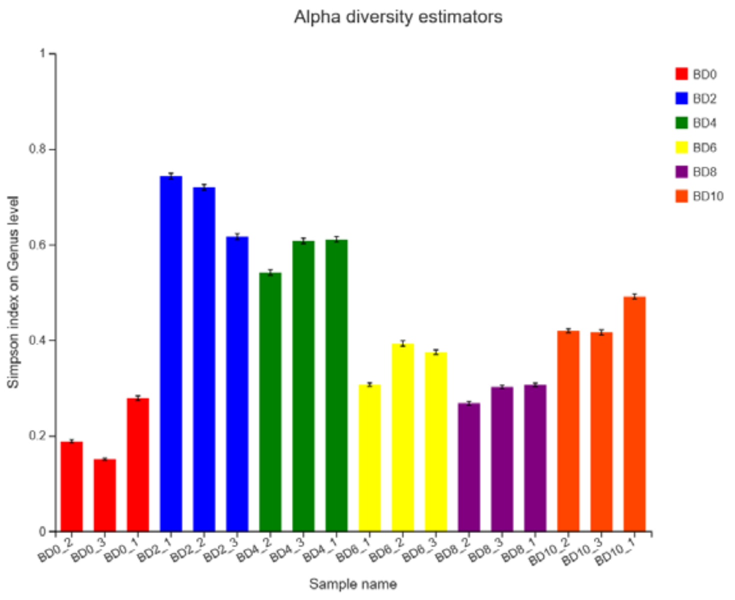

Figure S1. The fungal community diversity of the samples using alpha-diversity index.

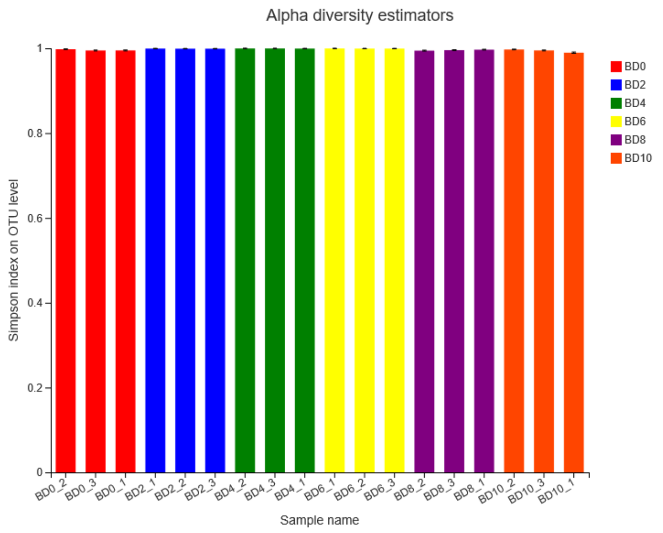

Figure S2. The bacterial community diversity of the samples using alpha-diversity index.

Table S1. The changes of alcohol, reducing sugar and acid during fermentation in northern Huangjiu.

	**Day 0**	**Day 2**	**Day 4**	**Day 6**	**Day 8**	**Day 10**
**Alcohol (%vol)**	0.000±0.000	6.520±0.347	11.646±0.962	12.132±0.408	12.298±0.303	12.584±0.345
**Reducing sugar (g/L)**	16.411±0.340	9.772±0.395	8.717±0.216	7.991±0.461	6.997±0.286	6.157±0.227
**Acid (g/L)**	0.837±0.052	3.736±0.263	5.255±0.229	5.093±0.775	5.007±0.184	5.013±0.425

*Every value was expressed as means standard error (n=3).

Table S2. The high-throughput sequencing of fungal diversity during the fermentation process in northern Huangjiu.

OTU ID	Day0_1	Day0_2	Day0_3	Day2_1	Day2_2	Day2_3	Day4_1	Day4_2	Day4_3
Aspergillus	8	6	6	1	0	2	0	0	1
Malassezia	1	1	1	0	0	2	1	4	2
Podosphaera	0	0	1	0	0	0	0	0	0
Saccharomyces	30339	32705	33521	31238	37968	38401	34770	41404	38034
Thermoascus	67	27	78	9	16	13	10	2	3

Refer to Table S2 (continued)

OTU ID	Day6_1	Day6_2	Day6_3	Day8_1	Day8_2	Day8_3	Day10_1	Day10_2	Day10_3
Aspergillus	2	0	1	0	1	1	1	0	2
Malassezia	0	1	0	3	1	1	3	1	1
Podosphaera	0	0	0	0	0	0	3	0	0
Saccharomyces	34125	37213	38754	35604	37345	38521	34253	35150	37495
Thermoascus	5	10	12	53	101	80	171	47	91

Table S3. The high-throughput sequencing of bacterial diversity during the fermentation process in northern Huangjiu.

OTU ID	BD0_1	BD0_2	BD0_3	BD2_1	BD2_2	BD2_3	BD4_1	BD4_2	BD4_3	BD6_1	BD6_2	BD6_3	BD8_1	BD8_2	BD8_3	BD10_1	BD10_2	BD10_3
*d__Bacteria;k__norank;p__Acidobacteria;c__Acidobacteria;o__Acidobacteriales;f__Acidobacteriaceae__Subgroup_1_;g__norank_f__Acidobacteriaceae__Subgroup_1_*	6	0	0	0	0	0	0	0	0	0	0	0	0	0	0	0	0	0
d__Bacteria;k__norank;p__Acidobacteria;c__Acidobacteria;o__Holophagales;f__Holophagaceae;g__norank_f__Holophagaceae	4	4	15	4	5	3	0	1	3	1	7	3	1	0	0	8	0	6
d__Bacteria;k__norank;p__Acidobacteria;c__Acidobacteria;o__norank_c__Acidobacteria;f__norank_c__Acidobacteria;g__norank_c__Acidobacteria	23	13	28	3	5	2	2	5	13	5	0	5	10	5	8	4	2	1
d__Bacteria;k__norank;p__Acidobacteria;c__Acidobacteria;o__Solibacterales;f__Solibacteraceae__Subgroup_3_;g__Bryobacter	5	0	3	0	0	0	0	0	2	1	0	1	0	0	12	0	0	0
d__Bacteria;k__norank;p__Acidobacteria;c__Acidobacteria;o__Solibacterales;f__Solibacteraceae__Subgroup_3_;g__Paludibaculum	3	0	2	3	0	2	2	0	0	0	0	0	2	0	0	11	0	0
d__Bacteria;k__norank;p__Acidobacteria;c__Acidobacteria;o__Subgroup_7;f__norank_o__Subgroup_7;g__norank_o__Subgroup_7	1	0	8	0	0	0	0	0	0	0	0	0	0	0	0	0	0	0
d__Bacteria;k__norank;p__Actinobacteria;c__Actinobacteria;o__Acidimicrobiales;f__Acidimicrobiaceae;g__CL500-29_marine_group	0	0	0	0	0	0	0	0	0	0	0	0	0	25	0	0	0	0
d__Bacteria;k__norank;p__Actinobacteria;c__Actinobacteria;o__Acidimicrobiales;f__Acidimicrobiaceae;g__Ilumatobacter	0	0	0	0	0	0	0	0	0	0	0	0	0	15	0	4	0	0
d__Bacteria;k__norank;p__Actinobacteria;c__Actinobacteria;o__Acidimicrobiales;f__Acidimicrobiaceae;g__norank_f__Acidimicrobiaceae	0	0	6	0	0	0	0	0	0	0	0	0	0	0	0	0	0	0

d__Bacteria;k__norank;p__Actinobacteria;c__Actinobacteria;o__Acidimicrobiales;f__norank_o__Acidimicrobiales;g__norank_o__Acidimicrobiales	1	0	0	0	1	0	0	0	0	1	0	0	1	0	0	4	0	7
d__Bacteria;k__norank;p__Actinobacteria;c__Actinobacteria;o__Actinomycetales;f__Actinomycetaceae;g__Actinomyces	4	2	22	1	0	7	3	0	1	0	26	1	2	1	1	1	4	21
d__Bacteria;k__norank;p__Actinobacteria;c__Actinobacteria;o__Actinopolysporales;f__Actinopolysporaceae;g__norank_f__Actinopolysporaceae	2	29	17	2	0	1	2	0	15	2	3	9	5	14	5	1	2	0
d__Bacteria;k__norank;p__Actinobacteria;c__Actinobacteria;o__Bifidobacteriales;f__Bifidobacteriaceae;g__Bifidobacterium	2	18	24	2	0	0	0	1	9	0	0	0	15	0	6	2	3	50
d__Bacteria;k__norank;p__Actinobacteria;c__Actinobacteria;o__Bifidobacteriales;f__Bifidobacteriaceae;g__Scardovia	0	0	0	0	0	0	0	0	0	0	0	0	0	0	0	0	0	24
d__Bacteria;k__norank;p__Actinobacteria;c__Actinobacteria;o__Coriobacteriales;f__Coriobacteriaceae;g__Enorma	0	0	3	0	0	0	0	0	0	0	0	0	0	0	0	0	0	0
d__Bacteria;k__norank;p__Actinobacteria;c__Actinobacteria;o__Coriobacteriales;f__Coriobacteriaceae;g__unclassified_f__Coriobacteriaceae	0	12	0	0	0	0	0	0	0	0	0	0	0	0	0	0	0	0
d__Bacteria;k__norank;p__Actinobacteria;c__Actinobacteria;o__Corynebacteriales;f__Corynebacteriaceae;g__Corynebacterium_1	4	38	45	4	8	9	3	6	13	24	26	28	3	5	28	9	28	53
d__Bacteria;k__norank;p__Actinobacteria;c__Actinobacteria;o__Corynebacteriales;f__Corynebacteriaceae;g__Lawsonella	8	11	13	0	0	1	0	2	0	4	3	1	1	1	3	3	2	0
d__Bacteria;k__norank;p__Actinobacteria;c__Actinobacteria;o__Corynebacteriales;f__Mycobacteriaceae;g__Mycobacterium	2	4	29	0	0	1	0	3	0	4	6	1	1	2	11	0	0	5
d__Bacteria;k__norank;p__Actinobacteria;c__Actinobacteria;o__Corynebacteriales;f__Nocardiaceae;g__Rhodococcus	494	626	902	78	108	84	119	81	146	364	1129	798	273	572	715	463	405	2224
d__Bacteria;k__norank;p__Actinobacteria;c__Actinobacteria;o__Frankiales;f__Acidothermaceae;g__Acidothermus	0	0	0	0	0	0	0	0	0	0	0	0	0	0	0	0	0	7

d__Bacteria;k__norank;p__Actinobacteria;c__Actinobacteria;o__Frankiales;f__Nakamurellaceae;g__Nakamurella	22	41	26	3	3	6	8	8	4	4	10	2	3	10	15	3	1	4
d__Bacteria;k__norank;p__Actinobacteria;c__Actinobacteria;o__Frankiales;f__Sporichthyaceae;g__hgcI_clade	0	0	0	0	0	0	0	0	0	0	0	0	0	10	0	0	0	0
d__Bacteria;k__norank;p__Actinobacteria;c__Actinobacteria;o__Gaiellales;f__Gaiellaceae;g__Gaiella	2	7	8	0	0	1	1	1	0	2	13	0	4	6	3	1	0	0
d__Bacteria;k__norank;p__Actinobacteria;c__Actinobacteria;o__Gaiellales;f__norank_o__Gaiellales;g__norank_o__Gaiellales	7	4	0	1	0	0	0	0	0	0	0	0	5	1	0	0	0	0
d__Bacteria;k__norank;p__Actinobacteria;c__Actinobacteria;o__Micrococcales;f__Brevibacteriaceae;g__Brevibacterium	0	0	0	0	0	0	0	0	0	1	0	0	0	0	0	0	0	19
d__Bacteria;k__norank;p__Actinobacteria;c__Actinobacteria;o__Micrococcales;f__Dermabacteraceae;g__Brachybacterium	8	1	0	0	0	0	2	0	1	0	0	6	3	5	0	0	0	0
d__Bacteria;k__norank;p__Actinobacteria;c__Actinobacteria;o__Micrococcales;f__Intrasporangiaceae;g__unclassified_f__Intrasporangiaceae	0	0	3	1	0	0	1	2	0	0	0	0	0	0	5	0	1	0
d__Bacteria;k__norank;p__Actinobacteria;c__Actinobacteria;o__Micrococcales;f__Microbacteriaceae;g__Microbacterium	4	2	2	3	4	1	2	3	1	1	0	1	8	0	1	3	2	6
d__Bacteria;k__norank;p__Actinobacteria;c__Actinobacteria;o__Micrococcales;f__Microbacteriaceae;g__Pseudoclavibacter	5	7	0	0	0	0	0	1	0	0	0	0	4	0	0	1	0	0
d__Bacteria;k__norank;p__Actinobacteria;c__Actinobacteria;o__Micrococcales;f__Microbacteriaceae;g__unclassified_f__Microbacteriaceae	0	2	0	0	0	0	0	0	0	0	0	0	0	0	0	0	0	0
d__Bacteria;k__norank;p__Actinobacteria;c__Actinobacteria;o__Micrococcales;f__Micrococcaceae;g__Kocuria	3	5	0	0	0	1	3	5	3	8	4	3	16	5	9	4	2	2
d__Bacteria;k__norank;p__Actinobacteria;c__Actinobacteria;o__Micrococcales;f__Micrococcaceae;g__Micrococcus	0	1	5	0	0	0	0	0	0	5	0	8	1	3	8	0	1	5

d__Bacteria;k__norank;p__Actinobacteria;c__Actinobacteria;o__Micrococcales;f__Micrococcaceae;g__Rothia	0	0	1	1	1	0	1	0	0	0	1	0	2	0	0	0	0	7
d__Bacteria;k__norank;p__Actinobacteria;c__Actinobacteria;o__Micrococcales;f__Promicromonosporaceae;g__Cellulosimicrobium	3	0	0	0	0	0	1	0	0	0	0	3	0	0	2	2	0	0
d__Bacteria;k__norank;p__Actinobacteria;c__Actinobacteria;o__PeM15;f__norank_o__PeM15;g__norank_o__PeM15	1	0	0	0	0	0	0	0	0	0	0	0	0	15	0	0	0	0
d__Bacteria;k__norank;p__Actinobacteria;c__Actinobacteria;o__Propionibacteriales;f__Nocardioidaceae;g__unclassified_f__Nocardioidaceae	0	0	9	0	0	0	0	0	0	0	0	0	0	0	0	0	0	0
d__Bacteria;k__norank;p__Actinobacteria;c__Actinobacteria;o__Propionibacteriales;f__Propionibacteriaceae;g__Microlunatus	1	4	0	0	0	0	0	1	0	0	0	0	1	1	0	0	0	0
d__Bacteria;k__norank;p__Actinobacteria;c__Actinobacteria;o__Propionibacteriales;f__Propionibacteriaceae;g__Propionibacterium	43	89	83	8	7	1	8	4	8	34	61	61	7	42	6	8	6	23
d__Bacteria;k__norank;p__Actinobacteria;c__Actinobacteria;o__Propionibacteriales;f__Propionibacteriaceae;g__Propioniciclava	0	0	0	0	0	0	0	0	0	0	0	0	0	0	0	0	0	15
d__Bacteria;k__norank;p__Actinobacteria;c__Actinobacteria;o__Pseudonocardiales;f__Pseudonocardiaceae;g__Pseudonocardia	0	0	0	0	0	0	0	0	0	2	0	0	0	0	0	0	0	0
d__Bacteria;k__norank;p__Actinobacteria;c__Actinobacteria;o__Pseudonocardiales;f__Pseudonocardiaceae;g__Saccharopolyspora	49	43	88	9	4	12	16	10	30	19	25	17	17	28	8	14	13	4
d__Bacteria;k__norank;p__Actinobacteria;c__Actinobacteria;o__Solirubrobacterales;f__Elev-16S-1332;g__norank_f__Elev-16S-1332	2	2	0	0	1	0	0	0	0	0	0	0	2	1	4	0	0	0
d__Bacteria;k__norank;p__Actinobacteria;c__Actinobacteria;o__Solirubrobacterales;f__Solirubrobacteraceae;g__Solirubrobacter	0	0	5	0	0	0	0	0	1	8	0	1	0	0	0	0	0	0
d__Bacteria;k__norank;p__Actinobacteria;c__Actinobacteria;o__Solirubrobacterales;f__unclassified_o__Solirubrobacterales;g__unclassified_o__Solirubrobacterales	0	0	7	0	0	0	0	0	0	0	0	0	0	0	0	0	0	0

d__Bacteria;k__norank;p__Actinobacteria;c__Actinobacteria;o__Streptomycetales;f__Streptomycetaceae;g__Streptomyces	1697	762	1348	151	129	149	308	167	260	430	824	705	564	587	659	341	301	288
d__Bacteria;k__norank;p__Bacteroidetes;c__Bacteroidetes_BD2-2;o__norank_c__Bacteroidetes_BD2-2;f__norank_c__Bacteroidetes_BD2-2;g__norank_c__Bacteroidetes_BD2-2	0	0	8	0	0	0	0	0	0	0	0	0	0	0	0	0	0	0
d__Bacteria;k__norank;p__Bacteroidetes;c__Bacteroidetes_VC2.1_Bac22;o__norank_c__Bacteroidetes_VC2.1_Bac22;f__norank_c__Bacteroidetes_VC2.1_Bac22;g__norank_c__Bacteroidetes_VC2.1_Bac22	3	0	4	0	0	0	0	0	0	0	0	0	0	0	0	0	0	0
d__Bacteria;k__norank;p__Bacteroidetes;c__Bacteroidia;o__Bacteroidales;f__Bacteroidaceae;g__Bacteroides	18	56	28	15	16	22	18	27	4	6	0	7	10	171	8	3	1	7
d__Bacteria;k__norank;p__Bacteroidetes;c__Bacteroidia;o__Bacteroidales;f__Bacteroidales_BS11_gut_group;g__norank_f__Bacteroidales_BS11_gut_group	10	55	11	9	3	8	4	4	3	2	22	11	4	15	0	3	5	1
d__Bacteria;k__norank;p__Bacteroidetes;c__Bacteroidia;o__Bacteroidales;f__Bacteroidales_S24-7_group;g__norank_f__Bacteroidales_S24-7_group	99	152	92	18	43	40	30	40	23	16	20	5	14	35	73	5	2	43
d__Bacteria;k__norank;p__Bacteroidetes;c__Bacteroidia;o__Bacteroidales;f__Bacteroidales_UCG-001;g__norank_f__Bacteroidales_UCG-001	0	6	0	0	0	0	0	0	1	3	0	0	0	0	0	0	0	0
d__Bacteria;k__norank;p__Bacteroidetes;c__Bacteroidia;o__Bacteroidales;f__Porphyromonadaceae;g__Odoribacter	2	5	8	1	4	1	0	5	0	0	1	0	0	0	0	0	0	0
d__Bacteria;k__norank;p__Bacteroidetes;c__Bacteroidia;o__Bacteroidales;f__Porphyromonadaceae;g__Parabacteroides	12	9	9	0	1	1	0	1	1	1	0	0	0	0	0	0	4	0

d__Bacteria;k__norank;p__Bacteroidetes;c__Bacteroidia;o__Bacteroidales;f__Porphyromonadaceae;g__Porphyromonas	4	2	2	7	1	1	0	0	5	0	22	1	2	1	1	3	2	0
d__Bacteria;k__norank;p__Bacteroidetes;c__Bacteroidia;o__Bacteroidales;f__Prevotellaceae;g__Alloprevotella	12	32	17	3	3	7	7	5	1	0	2	0	0	0	9	0	1	0
d__Bacteria;k__norank;p__Bacteroidetes;c__Bacteroidia;o__Bacteroidales;f__Prevotellaceae;g__Prevotella	0	0	0	0	1	0	0	0	0	2	0	4	0	0	1	3	5	4
d__Bacteria;k__norank;p__Bacteroidetes;c__Bacteroidia;o__Bacteroidales;f__Prevotellaceae;g__Prevotella_1	12	22	9	4	1	5	5	0	4	0	0	0	0	0	2	0	4	0
d__Bacteria;k__norank;p__Bacteroidetes;c__Bacteroidia;o__Bacteroidales;f__Prevotellaceae;g__Prevotella_7	0	18	13	1	3	12	1	1	2	2	13	1	5	0	12	0	1	20
d__Bacteria;k__norank;p__Bacteroidetes;c__Bacteroidia;o__Bacteroidales;f__Prevotellaceae;g__Prevotella_9	9	29	21	8	6	6	10	7	4	0	0	4	0	2	6	0	0	0
d__Bacteria;k__norank;p__Bacteroidetes;c__Bacteroidia;o__Bacteroidales;f__Prevotellaceae;g__Prevotellaceae_NK3B31_group	32	86	20	9	6	19	8	11	5	0	0	0	0	0	21	0	0	0
d__Bacteria;k__norank;p__Bacteroidetes;c__Bacteroidia;o__Bacteroidales;f__Prevotellaceae;g__Prevotellaceae_UCG-001	17	58	2	3	2	4	6	1	2	2	2	6	2	0	15	0	0	0
d__Bacteria;k__norank;p__Bacteroidetes;c__Bacteroidia;o__Bacteroidales;f__Prevotellaceae;g__Prevotellaceae_UCG-003	2	5	11	0	0	2	0	1	0	0	0	0	0	0	0	0	0	0
d__Bacteria;k__norank;p__Bacteroidetes;c__Bacteroidia;o__Bacteroidales;f__Prevotellaceae;g__unclassified_f__Prevotellaceae	19	11	0	3	1	1	2	0	0	0	0	0	0	0	0	0	0	0
d__Bacteria;k__norank;p__Bacteroidetes;c__Bacteroidia;o__Bacteroidales;f__Rikenellaceae;g__Alistipes	7	37	6	2	2	2	2	4	0	2	0	0	0	0	4	3	0	1
d__Bacteria;k__norank;p__Bacteroidetes;c__Bacteroidia;o__Bacteroidales;f__Rikenellaceae;g__Rikenellaceae_RC9_gut_group	7	34	18	2	4	7	4	2	0	0	0	1	0	14	3	2	0	2

d__Bacteria;k__norank;p__Bacteroidetes;c__Bacteroidia;o__Bacteroidales;f__Rikenellaceae;g__unclassified_f__Rikenellaceae	0	1	0	0	0	0	0	0	0	8	1	0	1	0	3	0	0	0
d__Bacteria;k__norank;p__Bacteroidetes;c__Cytophagia;o__Cytophagales;f__Cytophagaceae;g__Flectobacillus	0	0	0	0	0	0	0	0	0	0	0	0	0	0	5	0	0	0
d__Bacteria;k__norank;p__Bacteroidetes;c__Flavobacteriia;o__Flavobacteriales;f__Flavobacteriaceae;g__Capnocytophaga	0	1	0	0	0	0	1	1	0	0	14	2	0	0	0	1	0	0
d__Bacteria;k__norank;p__Bacteroidetes;c__Flavobacteriia;o__Flavobacteriales;f__Flavobacteriaceae;g__Chryseobacterium	0	0	0	0	0	0	0	3	0	2	7	2	0	1	0	8	1	0
d__Bacteria;k__norank;p__Bacteroidetes;c__Flavobacteriia;o__Flavobacteriales;f__Flavobacteriaceae;g__Cloacibacterium	0	8	0	2	0	0	0	1	0	2	9	1	3	2	8	11	1	0
d__Bacteria;k__norank;p__Bacteroidetes;c__Flavobacteriia;o__Flavobacteriales;f__Flavobacteriaceae;g__Spongiimonas	0	0	0	0	0	0	0	0	0	0	0	0	3	827	0	0	0	0
d__Bacteria;k__norank;p__Bacteroidetes;c__Flavobacteriia;o__Flavobacteriales;f__Flavobacteriaceae;g__unclassified_f__Flavobacteriaceae	0	0	0	0	0	0	0	0	0	0	0	0	5	12	0	0	0	0
d__Bacteria;k__norank;p__Bacteroidetes;c__Flavobacteriia;o__Flavobacteriales;f__NS9_marine_group;g__norank_f__NS9_marine_group	0	0	0	0	0	0	0	0	0	0	0	0	0	0	0	9	0	0
d__Bacteria;k__norank;p__Bacteroidetes;c__Sphingobacteriia;o__Sphingobacteriales;f__Chitinophagaceae;g__norank_f__Chitinophagaceae	6	0	13	0	0	0	0	2	0	0	0	0	0	1	0	0	0	0
d__Bacteria;k__norank;p__Bacteroidetes;c__Sphingobacteriia;o__Sphingobacteriales;f__Chitinophagaceae;g__Sediminibacterium	0	0	0	0	0	0	0	0	0	0	0	0	0	0	5	0	0	0
d__Bacteria;k__norank;p__Bacteroidetes;c__Sphingobacteriia;o__Sphingobacteriales;f__Chitinophagaceae;g__Vibrionimonas	6	0	3	0	3	1	1	0	0	0	17	0	0	1	4	1	0	0
d__Bacteria;k__norank;p__Bacteroidetes;c__Sphingobacteriia;o__Sphingobacteriales;f__PHOS-HE51;g__norank_f__PHOS-HE51	0	0	0	0	6	0	0	0	0	0	0	0	0	0	0	0	0	0

d__Bacteria;k__norank;p__Bacteroidetes;c__Sphingobacteriia;o__Sphingobacteriales;f__Sphingobacteriaceae;g__Sphingobacterium	0	0	0	0	0	0	0	0	0	0	0	0	0	0	0	0	0	17
d__Bacteria;k__norank;p__Chlamydiae;c__Chlamydiae;o__Chlamydiales;f__Simkaniaceae;g__norank_f__Simkaniaceae	0	1	0	0	0	0	0	0	0	0	1	0	0	0	0	0	1	0
d__Bacteria;k__norank;p__Chlamydiae;c__Chlamydiae;o__Chlamydiales;f__unclassified_o__Chlamydiales;g__unclassified_o__Chlamydiales	7	0	0	0	0	0	0	0	0	0	0	0	0	0	0	0	0	0
d__Bacteria;k__norank;p__Chlorobi;c__Chlorobia;o__Chlorobiales;f__OPB56;g__norank_f__OPB56	0	0	0	0	0	0	0	0	0	0	0	0	0	10	0	0	0	0
d__Bacteria;k__norank;p__Chloroflexi;c__Anaerolineae;o__Anaerolineales;f__Anaerolineaceae;g__norank_f__Anaerolineaceae	10	0	0	0	1	0	0	0	0	0	0	0	1	0	0	0	10	18
d__Bacteria;k__norank;p__Chloroflexi;c__Caldilineae;o__Caldilineales;f__Caldilineaceae;g__norank_f__Caldilineaceae	0	0	0	0	0	0	0	1	0	0	0	0	55	17	0	0	0	0
d__Bacteria;k__norank;p__Chloroflexi;c__SJA-15;o__norank_c__SJA-15;f__norank_c__SJA-15;g__norank_c__SJA-15	0	0	2	0	0	0	0	0	0	0	0	0	0	0	0	0	7	0
d__Bacteria;k__norank;p__Chloroflexi;c__Thermomicrobia;o__AKYG1722;f__norank_o__AKYG1722;g__norank_o__AKYG1722	6	0	0	0	0	0	0	0	0	0	0	0	0	0	0	0	0	0
d__Bacteria;k__norank;p__Chloroflexi;c__Thermomicrobia;o__JG30-KF-CM45;f__norank_o__JG30-KF-CM45;g__norank_o__JG30-KF-CM45	0	0	0	0	0	0	0	9	0	0	0	0	0	0	0	0	2	0
d__Bacteria;k__norank;p__Chloroflexi;c__Thermomicrobia;o__Sphaerobacterales;f__Sphaerobacteraceae;g__Nitrolancea	0	5	0	0	0	1	0	0	0	0	0	1	0	0	0	0	0	0
d__Bacteria;k__norank;p__Cyanobacteria;c__Cyanobacteria;o__norank_c__Cyanobacteria;f__norank_c__Cyanobacteria;g__norank_c__Cyanobacteria	21767	14128	11493	1603	2452	5492	4499	7888	5930	11656	6592	9486	13706	8951	####	8536	13806	7780

d__Bacteria;k__norank;p__Cyanobacteria;c__Cyanobacteria;o__Obscuribacterales;f__norank_o__Obscuribacterales;g__norank_o__Obscuribacterales	15	0	4	0	1	0	1	0	0	0	0	2	0	0	0	2	0	1
d__Bacteria;k__norank;p__Cyanobacteria;c__Cyanobacteria;o__SubsectionI;f__FamilyI_o__SubsectionI;g__norank_f__FamilyI	0	0	0	0	0	0	0	1	0	0	4	0	9	0	0	0	0	0
d__Bacteria;k__norank;p__Cyanobacteria;c__Cyanobacteria;o__SubsectionI;f__FamilyI_o__SubsectionI;g__Synechococcus	0	4	0	0	0	0	0	5	0	0	0	0	8	141	0	0	0	0
d__Bacteria;k__norank;p__Cyanobacteria;c__Cyanobacteria;o__SubsectionI;f__FamilyI_o__SubsectionI;g__unclassified_f__FamilyI_o__SubsectionI	0	0	0	0	0	0	0	0	0	0	0	0	14	373	0	0	0	0
d__Bacteria;k__norank;p__Cyanobacteria;c__Cyanobacteria;o__SubsectionII;f__FamilyII_o__SubsectionII;g__Chroococcidiopsis	0	0	9	0	0	0	0	0	0	0	0	4	0	0	0	0	0	0
d__Bacteria;k__norank;p__Cyanobacteria;c__Cyanobacteria;o__SubsectionIII;f__FamilyI_o__SubsectionIII;g__unclassified_f__FamilyI_o__SubsectionIII	0	0	0	0	0	0	0	0	0	0	0	5	2	20	0	0	0	0
d__Bacteria;k__norank;p__Deinococcus-Thermus;c__Deinococci;o__Deinococcales;f__Deinococcaceae;g__Deinococcus	0	4	0	1	0	0	0	0	0	0	0	3	2	8	7	8	0	0
d__Bacteria;k__norank;p__Deinococcus-Thermus;c__Deinococci;o__Deinococcales;f__Trueperaceae;g__Truepera	0	0	0	0	0	1	0	13	0	0	0	0	0	0	0	0	0	0
d__Bacteria;k__norank;p__Deinococcus-Thermus;c__Deinococci;o__Thermales;f__Thermaceae;g__Thermus	0	0	0	0	0	0	1	0	0	0	0	0	0	11	0	0	0	0
d__Bacteria;k__norank;p__Firmicutes;c__Bacilli;o__Bacillales;f__Bacillaceae;g__Bacillus	1380	301	398	44	32	47	63	51	52	165	193	184	120	151	269	93	55	72
d__Bacteria;k__norank;p__Firmicutes;c__Bacilli;o__Bacillales;f__Bacillaceae;g__Geobacillus	0	0	0	0	1	0	1	0	1	3	6	2	2	1	0	0	2	0

d__Bacteria;k__norank;p__Firmicutes;c__Bacilli;o__Bacillales;f__Bacillaceae;g__Oceanobacillus	37	22	27	5	0	9	6	8	8	5	20	15	5	2	0	7	1	4
d__Bacteria;k__norank;p__Firmicutes;c__Bacilli;o__Bacillales;f__Bacillaceae;g__Thalassobacillus	1	0	9	0	0	0	0	0	0	0	0	0	0	0	0	0	0	0
d__Bacteria;k__norank;p__Firmicutes;c__Bacilli;o__Bacillales;f__Bacillaceae;g__unclassified_f__Bacillaceae	0	0	0	0	0	0	0	0	1	0	0	0	5	0	0	0	0	0
d__Bacteria;k__norank;p__Firmicutes;c__Bacilli;o__Bacillales;f__Bacillaceae;g__Virgibacillus	0	5	0	0	3	0	2	0	5	0	3	7	3	0	0	5	0	10
d__Bacteria;k__norank;p__Firmicutes;c__Bacilli;o__Bacillales;f__Family_XI_o__Bacillales;g__Gemella	1	4	0	0	0	0	1	0	0	0	9	2	1	1	1	3	0	0
d__Bacteria;k__norank;p__Firmicutes;c__Bacilli;o__Bacillales;f__Paenibacillaceae;g__Paenibacillus	20	69	27	7	6	12	12	4	1	12	49	11	7	9	9	5	5	1
d__Bacteria;k__norank;p__Firmicutes;c__Bacilli;o__Bacillales;f__Planococcaceae;g__Lysinibacillus	0	1	7	0	0	0	0	0	0	0	0	0	2	4	0	0	0	4
d__Bacteria;k__norank;p__Firmicutes;c__Bacilli;o__Bacillales;f__Planococcaceae;g__Solibacillus	11	9	10	2	2	2	2	2	0	9	8	6	0	7	1	7	6	0
d__Bacteria;k__norank;p__Firmicutes;c__Bacilli;o__Bacillales;f__Staphylococcaceae;g__Staphylococcus	6	23	25	5	8	3	4	5	0	4	12	11	4	13	7	5	10	44
d__Bacteria;k__norank;p__Firmicutes;c__Bacilli;o__Bacillales;f__Thermoactinomycetaceae;g__Kroppenstedtia	383	631	432	79	30	39	81	70	84	118	286	175	91	218	172	89	82	51
d__Bacteria;k__norank;p__Firmicutes;c__Bacilli;o__Bacillales;f__Thermoactinomycetaceae;g__Thermoactinomyces	0	0	9	0	0	0	0	2	2	1	1	1	0	6	1	0	1	0
d__Bacteria;k__norank;p__Firmicutes;c__Bacilli;o__Lactobacillales;f__Aerococcaceae;g__Abiotrophia	0	0	0	0	0	0	0	0	0	0	11	0	3	0	0	2	0	0

d__Bacteria;k__norank;p__Firmicutes;c__Bacilli;o__Lactobacillales;f__Carnobacteriaceae;g__Granulicatella	0	0	2	0	1	0	0	2	3	0	1	0	12	4	0	2	1	7
d__Bacteria;k__norank;p__Firmicutes;c__Bacilli;o__Lactobacillales;f__Enterococcaceae;g__Enterococcus	40	49	108	15	6	23	5	8	7	4	10	23	11	3	0	10	15	0
d__Bacteria;k__norank;p__Firmicutes;c__Bacilli;o__Lactobacillales;f__Lactobacillaceae;g__Lactobacillus	40	72	41	5	154	104	140	25	5	3	118	101	131	35	29	5	7	1
d__Bacteria;k__norank;p__Firmicutes;c__Bacilli;o__Lactobacillales;f__Lactobacillaceae;g__Pediococcus	32	35	46	28	30	50	50	118	117	153	106	113	607	752	568	111	104	49
d__Bacteria;k__norank;p__Firmicutes;c__Bacilli;o__Lactobacillales;f__Leuconostocaceae;g__Leuconostoc	5	14	16	0	5	1	32	2	4	2	10	0	7	1	0	2	2	1
d__Bacteria;k__norank;p__Firmicutes;c__Bacilli;o__Lactobacillales;f__Leuconostocaceae;g__Weissella	56	102	51	105	57	57	223	82	58	86	141	71	51	24	47	30	8	44
d__Bacteria;k__norank;p__Firmicutes;c__Bacilli;o__Lactobacillales;f__Streptococcaceae;g__Lactococcus	4425	4288	7896	####	####	34860	37187	36753	33139	16018	33808	26601	20142	####	####	####	26255	24481
d__Bacteria;k__norank;p__Firmicutes;c__Bacilli;o__Lactobacillales;f__Streptococcaceae;g__Streptococcus	31	34	86	4	6	4	10	20	16	15	51	11	6	13	28	8	20	20
d__Bacteria;k__norank;p__Firmicutes;c__Clostridia;o__Clostridiales;f__Christensenellaceae;g__Christensenellaceae_R-7_group	8	10	6	2	0	1	1	0	0	0	7	3	0	1	2	1	2	0
d__Bacteria;k__norank;p__Firmicutes;c__Clostridia;o__Clostridiales;f__Clostridiaceae_1;g__Clostridium_sensu_stricto_1	7	6	6	1	0	0	9	4	10	5	4	6	2	0	3	1	4	3
d__Bacteria;k__norank;p__Firmicutes;c__Clostridia;o__Clostridiales;f__Clostridiaceae_1;g__Clostridium_sensu_stricto_13	0	0	0	0	0	0	0	0	0	0	0	0	0	0	5	0	3	0
d__Bacteria;k__norank;p__Firmicutes;c__Clostridia;o__Clostridiales;f__Clostridiaceae_1;g__Clostridium_sensu_stricto_15	4	0	0	0	0	1	0	0	0	0	0	0	0	0	0	0	0	0

d__Bacteria;k__norank;p__Firmicutes;c__Clostridia;o__Clostridiales;f__Clostridiales_vadinBB60_group;g__norank_f__Clostridiales_vadinBB60_group	0	10	0	0	0	0	0	0	0	0	0	0	0	0	0	0	0	0
d__Bacteria;k__norank;p__Firmicutes;c__Clostridia;o__Clostridiales;f__Family_XI_o__Clostridiales;g__Anaerococcus	0	0	0	0	2	0	0	0	0	2	0	0	0	0	3	1	1	14
d__Bacteria;k__norank;p__Firmicutes;c__Clostridia;o__Clostridiales;f__Family_XI_o__Clostridiales;g__Ezakiella	0	0	0	0	0	0	0	0	0	0	0	0	0	0	8	0	0	0
d__Bacteria;k__norank;p__Firmicutes;c__Clostridia;o__Clostridiales;f__Family_XI_o__Clostridiales;g__Finegoldia	0	0	2	0	0	1	0	1	0	0	4	5	5	0	0	5	4	15
d__Bacteria;k__norank;p__Firmicutes;c__Clostridia;o__Clostridiales;f__Family_XI_o__Clostridiales;g__Gallicola	0	0	0	0	0	0	0	0	0	0	0	0	29	0	0	0	0	0
d__Bacteria;k__norank;p__Firmicutes;c__Clostridia;o__Clostridiales;f__Family_XI_o__Clostridiales;g__Peptoniphilus	0	4	4	0	0	0	0	0	0	0	7	2	13	0	4	1	2	3
d__Bacteria;k__norank;p__Firmicutes;c__Clostridia;o__Clostridiales;f__Family_XI_o__Clostridiales;g__Sedimentibacter	0	10	0	0	0	0	0	0	0	0	0	0	0	0	0	0	0	0
d__Bacteria;k__norank;p__Firmicutes;c__Clostridia;o__Clostridiales;f__Family_XIII;g__[Eubacterium]_nodatum_group	0	0	0	2	0	1	0	1	0	0	0	0	0	0	0	0	0	0
d__Bacteria;k__norank;p__Firmicutes;c__Clostridia;o__Clostridiales;f__Family_XIII;g__Family_XIII_AD3011_group	0	8	4	1	3	0	0	0	0	0	2	0	0	0	0	0	0	0
d__Bacteria;k__norank;p__Firmicutes;c__Clostridia;o__Clostridiales;f__Family_XIII;g__norank_f__XIII	0	5	0	0	0	0	0	0	0	0	0	0	0	0	0	0	0	0
d__Bacteria;k__norank;p__Firmicutes;c__Clostridia;o__Clostridiales;f__Lachnospiraceae;g__[Eubacterium]_eligens_group	1	1	3	0	0	4	1	1	0	0	0	0	0	0	0	0	0	0
d__Bacteria;k__norank;p__Firmicutes;c__Clostridia;o__Clostridiales;f__Lachnospiraceae;g__[Eubacterium]_fissicatena_group	1	5	0	0	1	2	1	4	1	3	3	1	0	0	0	1	0	0

d__Bacteria;k__norank;p__Firmicutes;c__Clostridia;o__Clostridiales;f__Lachnospiraceae;g__[Eubacterium]_hallii_group	0	0	0	0	0	0	0	0	0	0	3	0	0	0	0	0	0	0
d__Bacteria;k__norank;p__Firmicutes;c__Clostridia;o__Clostridiales;f__Lachnospiraceae;g__[Ruminococcus]_gnavus_group	0	2	6	1	0	2	1	0	1	0	0	0	12	0	1	3	0	0
d__Bacteria;k__norank;p__Firmicutes;c__Clostridia;o__Clostridiales;f__Lachnospiraceae;g__[Ruminococcus]_torques_group	7	1	2	0	0	13	2	0	0	0	3	2	0	0	0	0	0	0
d__Bacteria;k__norank;p__Firmicutes;c__Clostridia;o__Clostridiales;f__Lachnospiraceae;g__Anaerostipes	2	6	4	0	1	2	0	5	0	0	0	0	0	0	2	0	0	0
d__Bacteria;k__norank;p__Firmicutes;c__Clostridia;o__Clostridiales;f__Lachnospiraceae;g__Blautia	9	0	3	0	0	0	0	0	1	0	0	1	0	0	5	0	0	1
d__Bacteria;k__norank;p__Firmicutes;c__Clostridia;o__Clostridiales;f__Lachnospiraceae;g__Coprococcus_1	0	0	5	0	0	0	1	1	0	0	4	0	0	0	0	0	0	0
d__Bacteria;k__norank;p__Firmicutes;c__Clostridia;o__Clostridiales;f__Lachnospiraceae;g__Coprococcus_3	0	0	7	1	0	1	2	0	0	0	0	1	0	0	0	0	0	0
d__Bacteria;k__norank;p__Firmicutes;c__Clostridia;o__Clostridiales;f__Lachnospiraceae;g__Lachnoanaerobaculum	0	0	0	0	0	0	2	0	0	0	3	1	0	0	0	0	1	15
d__Bacteria;k__norank;p__Firmicutes;c__Clostridia;o__Clostridiales;f__Lachnospiraceae;g__Lachnoclostridium	0	0	0	0	0	2	0	0	0	2	0	0	7	0	0	0	0	0
d__Bacteria;k__norank;p__Firmicutes;c__Clostridia;o__Clostridiales;f__Lachnospiraceae;g__Lachnospira	6	0	2	0	0	0	0	1	0	0	7	0	0	0	0	0	0	0
d__Bacteria;k__norank;p__Firmicutes;c__Clostridia;o__Clostridiales;f__Lachnospiraceae;g__Lachnospiraceae_NK4A136_group	14	8	17	6	12	8	8	8	6	0	0	0	0	1	11	0	2	0
d__Bacteria;k__norank;p__Firmicutes;c__Clostridia;o__Clostridiales;f__Lachnospiraceae;g__Lachnospiraceae_UCG-001	1	0	0	0	0	1	1	0	2	0	0	0	0	0	5	0	0	0

d__Bacteria;k__norank;p__Firmicutes;c__Clostridia;o__Clostridiales;f__Lachnospiraceae;g__Lachnospiraceae_UCG-010	0	0	5	0	0	0	0	0	0	0	0	0	0	0	0	0	0	0
d__Bacteria;k__norank;p__Firmicutes;c__Clostridia;o__Clostridiales;f__Lachnospiraceae;g__Marvinbryantia	1	5	0	0	0	0	0	0	0	1	1	0	0	0	0	0	0	2
d__Bacteria;k__norank;p__Firmicutes;c__Clostridia;o__Clostridiales;f__Lachnospiraceae;g__norank_f__Lachnospiraceae	7	23	20	1	5	2	8	2	0	5	2	3	0	2	23	0	0	0
d__Bacteria;k__norank;p__Firmicutes;c__Clostridia;o__Clostridiales;f__Lachnospiraceae;g__Oribacterium	0	0	4	0	0	1	1	0	0	0	0	0	0	0	0	0	0	0
d__Bacteria;k__norank;p__Firmicutes;c__Clostridia;o__Clostridiales;f__Lachnospiraceae;g__Roseburia	3	21	5	1	2	4	4	7	3	0	0	1	0	3	35	0	1	0
d__Bacteria;k__norank;p__Firmicutes;c__Clostridia;o__Clostridiales;f__Lachnospiraceae;g__Tyzzerella_3	0	0	10	0	0	0	0	0	0	0	0	0	0	0	0	0	0	0
d__Bacteria;k__norank;p__Firmicutes;c__Clostridia;o__Clostridiales;f__Lachnospiraceae;g__unclassified_f__Lachnospiraceae	5	34	12	0	3	11	3	4	1	3	2	0	0	0	34	2	0	0
d__Bacteria;k__norank;p__Firmicutes;c__Clostridia;o__Clostridiales;f__Peptostreptococcaceae;g__Filifactor	1	0	0	0	0	0	0	0	0	0	0	0	1	1	0	1	3	0
d__Bacteria;k__norank;p__Firmicutes;c__Clostridia;o__Clostridiales;f__Peptostreptococcaceae;g__Peptoclostridium	0	5	0	0	0	0	2	0	3	0	0	0	0	0	2	0	0	0
d__Bacteria;k__norank;p__Firmicutes;c__Clostridia;o__Clostridiales;f__Peptostreptococcaceae;g__Romboutsia	12	3	6	7	0	3	5	7	4	6	8	1	0	10	2	2	4	0
d__Bacteria;k__norank;p__Firmicutes;c__Clostridia;o__Clostridiales;f__Ruminococcaceae;g__[Eubacterium]_coprostanoligenes_group	10	30	14	3	2	7	6	0	1	0	11	0	0	0	13	0	0	0
d__Bacteria;k__norank;p__Firmicutes;c__Clostridia;o__Clostridiales;f__Ruminococcaceae;g__Anaerofilum	0	0	0	0	0	9	0	0	0	0	0	0	0	0	0	0	0	0

d__Bacteria;k__norank;p__Firmicutes;c__Clostridia;o__Clostridiales;f__Ruminococcaceae;g__Anaerotruncus	3	3	1	2	2	3	0	4	0	0	0	0	0	0	0	0	0	0
d__Bacteria;k__norank;p__Firmicutes;c__Clostridia;o__Clostridiales;f__Ruminococcaceae;g__Faecalibacterium	14	26	27	9	4	6	9	8	1	2	3	3	3	7	7	9	2	2
d__Bacteria;k__norank;p__Firmicutes;c__Clostridia;o__Clostridiales;f__Ruminococcaceae;g__Fastidiosipila	0	0	0	0	0	0	0	0	0	0	0	0	6	0	0	0	0	0
d__Bacteria;k__norank;p__Firmicutes;c__Clostridia;o__Clostridiales;f__Ruminococcaceae;g__Oscillibacter	0	8	0	0	0	1	0	3	0	0	0	0	0	0	0	0	0	0
d__Bacteria;k__norank;p__Firmicutes;c__Clostridia;o__Clostridiales;f__Ruminococcaceae;g__Papillibacter	3	0	0	0	0	0	0	0	0	0	0	0	0	0	0	0	0	0
d__Bacteria;k__norank;p__Firmicutes;c__Clostridia;o__Clostridiales;f__Ruminococcaceae;g__Ruminiclostridium	0	11	0	1	0	2	2	1	3	0	0	0	1	0	4	0	0	0
d__Bacteria;k__norank;p__Firmicutes;c__Clostridia;o__Clostridiales;f__Ruminococcaceae;g__Ruminiclostridium_1	0	0	0	0	0	0	0	0	0	0	0	0	0	0	8	0	0	0
d__Bacteria;k__norank;p__Firmicutes;c__Clostridia;o__Clostridiales;f__Ruminococcaceae;g__Ruminiclostridium_9	1	21	4	1	5	2	2	2	1	0	0	0	0	0	2	0	1	18
d__Bacteria;k__norank;p__Firmicutes;c__Clostridia;o__Clostridiales;f__Ruminococcaceae;g__Ruminococcaceae_NK4A214_group	8	0	6	2	1	2	2	5	1	0	0	0	5	0	15	3	0	0
d__Bacteria;k__norank;p__Firmicutes;c__Clostridia;o__Clostridiales;f__Ruminococcaceae;g__Ruminococcaceae_UCG-002	8	21	3	6	4	7	6	3	1	0	6	1	0	1	8	1	3	0
d__Bacteria;k__norank;p__Firmicutes;c__Clostridia;o__Clostridiales;f__Ruminococcaceae;g__Ruminococcaceae_UCG-005	6	13	7	6	1	4	0	1	1	0	0	0	0	0	16	0	0	0
d__Bacteria;k__norank;p__Firmicutes;c__Clostridia;o__Clostridiales;f__Ruminococcaceae;g__Ruminococcaceae_UCG-007	0	0	11	0	0	0	0	0	0	0	0	0	0	0	0	0	0	0

d__Bacteria;k__norank;p__Firmicutes;c__Clostridia;o__Clostridiales;f__Ruminococcaceae;g__Ruminococcaceae_UCG-010	5	0	0	0	0	0	0	0	0	0	0	0	0	0	0	0	0	0
d__Bacteria;k__norank;p__Firmicutes;c__Clostridia;o__Clostridiales;f__Ruminococcaceae;g__Ruminococcaceae_UCG-011	1	3	12	0	1	0	0	1	0	2	4	0	0	0	3	0	0	0
d__Bacteria;k__norank;p__Firmicutes;c__Clostridia;o__Clostridiales;f__Ruminococcaceae;g__Ruminococcaceae_UCG-014	1	11	0	1	6	4	5	1	2	0	1	0	0	0	9	0	0	0
d__Bacteria;k__norank;p__Firmicutes;c__Clostridia;o__Clostridiales;f__Ruminococcaceae;g__Ruminococcus_1	0	25	4	0	0	1	1	0	0	2	0	0	0	0	1	0	0	0
d__Bacteria;k__norank;p__Firmicutes;c__Clostridia;o__Clostridiales;f__Ruminococcaceae;g__Ruminococcus_2	0	17	11	2	0	2	4	1	0	9	0	0	2	0	0	1	0	0
d__Bacteria;k__norank;p__Firmicutes;c__Clostridia;o__Clostridiales;f__Ruminococcaceae;g__Subdoligranulum	6	16	11	1	0	6	2	3	0	1	0	0	0	0	3	0	0	0
d__Bacteria;k__norank;p__Firmicutes;c__Erysipelotrichia;o__Erysipelotrichales;f__Erysipelotrichaceae;g__Catenibacterium	0	0	6	0	0	1	1	0	0	0	0	0	0	0	0	0	0	0
d__Bacteria;k__norank;p__Firmicutes;c__Erysipelotrichia;o__Erysipelotrichales;f__Erysipelotrichaceae;g__Erysipelatoclostridium	0	0	4	2	1	3	0	3	0	0	3	0	0	0	0	4	3	0
d__Bacteria;k__norank;p__Firmicutes;c__Erysipelotrichia;o__Erysipelotrichales;f__Erysipelotrichaceae;g__Erysipelotrichaceae_UCG-002	6	0	0	1	0	1	0	1	0	0	0	0	0	0	0	0	0	3
d__Bacteria;k__norank;p__Firmicutes;c__Erysipelotrichia;o__Erysipelotrichales;f__Erysipelotrichaceae;g__Faecalibaculum	2	12	11	0	0	0	1	6	2	2	6	4	5	1	1	1	0	0
d__Bacteria;k__norank;p__Firmicutes;c__Erysipelotrichia;o__Erysipelotrichales;f__Erysipelotrichaceae;g__Holdemanella	11	0	0	1	0	0	2	1	0	0	0	0	0	0	0	0	0	0
d__Bacteria;k__norank;p__Firmicutes;c__Erysipelotrichia;o__Erysipelotrichales;f__Erysipelotrichaceae;g__norank_f__Erysipelotrichaceae	1	9	8	0	4	7	4	14	0	0	0	0	4	1	0	0	6	0

d__Bacteria;k__norank;p__Firmicutes;c__Limnochordia;o__Limnochordales;f__norank_o__Limnochordales;g__norank_o__Limnochordales	17	0	0	0	0	0	0	0	0	0	0	0	0	0	0	0	0	0
d__Bacteria;k__norank;p__Firmicutes;c__Negativicutes;o__Selenomonadales;f__Acidaminococcaceae;g__Phascolarctobacterium	8	8	6	2	1	1	0	3	1	0	0	0	1	0	10	0	0	0
d__Bacteria;k__norank;p__Firmicutes;c__Negativicutes;o__Selenomonadales;f__Veillonellaceae;g__Dialister	4	12	0	0	0	0	1	2	1	0	0	0	0	0	0	0	2	0
d__Bacteria;k__norank;p__Firmicutes;c__Negativicutes;o__Selenomonadales;f__Veillonellaceae;g__Megasphaera	13	16	17	0	0	2	1	7	0	0	0	2	2	0	19	0	0	0
d__Bacteria;k__norank;p__Firmicutes;c__Negativicutes;o__Selenomonadales;f__Veillonellaceae;g__Schwartzia	0	0	0	0	0	0	0	0	0	0	0	0	0	0	0	0	0	29
d__Bacteria;k__norank;p__Firmicutes;c__Negativicutes;o__Selenomonadales;f__Veillonellaceae;g__Selenomonas	3	12	6	0	0	3	0	1	0	0	0	1	0	1	1	0	0	0
d__Bacteria;k__norank;p__Firmicutes;c__Negativicutes;o__Selenomonadales;f__Veillonellaceae;g__Veillonella	3	0	5	0	1	1	0	0	1	12	14	3	10	1	1	2	3	9
d__Bacteria;k__norank;p__Fusobacteria;c__Fusobacteriia;o__Fusobacteriales;f__Fusobacteriaceae;g__Cetobacterium	0	13	0	0	4	2	1	9	1	8	23	0	2	0	7	1	0	0
d__Bacteria;k__norank;p__Fusobacteria;c__Fusobacteriia;o__Fusobacteriales;f__Fusobacteriaceae;g__Fusobacterium	2	0	19	0	0	3	1	0	3	0	21	7	7	2	0	1	2	31
d__Bacteria;k__norank;p__Fusobacteria;c__Fusobacteriia;o__Fusobacteriales;f__Fusobacteriaceae;g__Propionigenium	0	0	0	0	0	0	0	0	0	0	0	0	0	10	0	0	0	0
d__Bacteria;k__norank;p__Fusobacteria;c__Fusobacteriia;o__Fusobacteriales;f__Leptotrichiaceae;g__Leptotrichia	0	2	33	1	0	5	0	0	2	0	20	6	12	0	2	0	4	5
d__Bacteria;k__norank;p__Fusobacteria;c__Fusobacteriia;o__Fusobacteriales;f__Leptotrichiaceae;g__norank_f__Leptotrichiaceae	0	0	0	0	0	0	0	0	0	0	0	0	0	80	0	0	0	0

d__Bacteria;k__norank;p__Gemmatimonadetes;c__Gemmatimonadetes;o__Gemmatimonadales;f__Gemmatimonadaceae;g__norank_f__Gemmatimonadaceae	0	0	7	0	0	0	0	0	0	0	0	0	0	0	0	0	0	0
d__Bacteria;k__norank;p__Gemmatimonadetes;c__Gemmatimonadetes;o__Longimicrobiales;f__Longimicrobiaceae;g__norank_f__Longimicrobiaceae	0	0	0	0	0	0	0	0	0	0	5	0	0	0	0	0	0	7
d__Bacteria;k__norank;p__Latescibacteria;c__norank_p__Latescibacteria;o__norank_p__Latescibacteria;f__norank_p__Latescibacteria;g__norank_p__Latescibacteria	0	0	0	0	0	0	0	0	0	0	0	0	0	0	8	0	0	0
d__Bacteria;k__norank;p__Nitrospirae;c__Nitrospira;o__norank_c__Nitrospira;f__norank_c__Nitrospira;g__Nitrospira	25	24	34	9	7	10	7	3	6	13	14	17	3	4	8	11	12	8
d__Bacteria;k__norank;p__Omnitrophica;c__norank_p__Omnitrophica;o__norank_p__Omnitrophica;f__norank_p__Omnitrophica;g__norank_p__Omnitrophica	0	0	0	0	0	0	0	0	1	2	0	0	1	1	0	0	0	0
d__Bacteria;k__norank;p__Parcubacteria;c__norank_p__Parcubacteria;o__norank_p__Parcubacteria;f__norank_p__Parcubacteria;g__norank_p__Parcubacteria	0	8	5	0	0	0	2	2	0	4	0	0	0	0	0	2	0	2
d__Bacteria;k__norank;p__Planctomycetes;c__BD7-11;o__norank_c__BD7-11;f__norank_c__BD7-11;g__norank_c__BD7-11	11	3	14	2	2	3	0	2	1	3	2	2	7	0	1	1	3	0
d__Bacteria;k__norank;p__Planctomycetes;c__Phycisphaerae;o__Phycisphaerales;f__Phycisphaeraceae;g__CL500-3	0	0	0	0	0	0	0	0	0	0	0	0	0	21	0	0	0	0
d__Bacteria;k__norank;p__Planctomycetes;c__Planctomycetacia;o__Brocadiales;f__Brocadiaceae;g__Candidatus_Kuenenia	2	0	0	0	0	0	0	0	0	0	0	0	0	0	0	0	4	17
d__Bacteria;k__norank;p__Planctomycetes;c__vadinHA49;o__norank_c__vadinHA49;f__norank_c__vadinHA49;g__norank_c__vadinHA49	0	0	0	0	0	0	0	0	0	0	0	0	5	0	0	0	0	0

d__Bacteria;k__norank;p__Proteobacteria;c__Alphaproteobacteria;o__Caulobacterales;f__Caulobacteraceae;g__Brevundimonas	89	90	107	19	9	24	47	25	12	51	125	31	42	36	75	35	51	31
d__Bacteria;k__norank;p__Proteobacteria;c__Alphaproteobacteria;o__Caulobacterales;f__Caulobacteraceae;g__Caulobacter	2	1	0	2	0	0	0	0	0	0	9	0	1	2	0	1	0	0
d__Bacteria;k__norank;p__Proteobacteria;c__Alphaproteobacteria;o__Caulobacterales;f__Caulobacteraceae;g__norank_f__Caulobacteraceae	1	1	0	1	2	1	0	1	0	0	0	3	0	0	0	0	1	0
d__Bacteria;k__norank;p__Proteobacteria;c__Alphaproteobacteria;o__Caulobacterales;f__Hyphomonadaceae;g__Hirschia	0	0	0	0	0	0	0	0	0	0	0	0	0	11	0	0	0	0
d__Bacteria;k__norank;p__Proteobacteria;c__Alphaproteobacteria;o__Caulobacterales;f__Hyphomonadaceae;g__Woodsholea	0	0	0	0	0	0	0	0	0	0	0	0	0	0	0	0	0	21
d__Bacteria;k__norank;p__Proteobacteria;c__Alphaproteobacteria;o__Rhizobiales;f__Bradyrhizobiaceae;g__Bosea	1	0	0	1	1	2	4	0	0	5	8	0	3	3	4	2	2	0
d__Bacteria;k__norank;p__Proteobacteria;c__Alphaproteobacteria;o__Rhizobiales;f__Bradyrhizobiaceae;g__Bradyrhizobium	4	14	19	3	6	4	7	2	0	1	17	17	4	40	10	11	9	25
d__Bacteria;k__norank;p__Proteobacteria;c__Alphaproteobacteria;o__Rhizobiales;f__Bradyrhizobiaceae;g__Rhodopseudomonas	1	7	1	0	1	1	0	0	0	0	1	3	0	7	0	1	0	0
d__Bacteria;k__norank;p__Proteobacteria;c__Alphaproteobacteria;o__Rhizobiales;f__Brucellaceae;g__Ochrobactrum	74	35	53	12	19	21	23	17	11	21	127	49	14	7	12	21	11	11
d__Bacteria;k__norank;p__Proteobacteria;c__Alphaproteobacteria;o__Rhizobiales;f__Hyphomicrobiaceae;g__Devosia	9	0	9	1	0	0	0	1	0	0	0	1	0	0	0	1	0	0
d__Bacteria;k__norank;p__Proteobacteria;c__Alphaproteobacteria;o__Rhizobiales;f__Hyphomicrobiaceae;g__Pedomicrobium	1	0	0	0	1	0	1	0	0	1	3	0	0	0	7	0	0	0
d__Bacteria;k__norank;p__Proteobacteria;c__Alphaproteobacteria;o__Rhizobiales;f__Methylobacteriaceae;g__Methylobacterium	4	0	5	0	1	0	0	0	2	0	13	9	1	0	1	0	2	15

d__Bacteria;k__norank;p__Proteobacteria;c__Alphaproteobacteria;o__Rhizobiales;f__Methylobacteriaceae;g__Microvirga	0	0	2	0	0	2	0	0	0	0	0	0	1	0	2	0	0	3
d__Bacteria;k__norank;p__Proteobacteria;c__Alphaproteobacteria;o__Rhizobiales;f__MNG7;g__norank_f__MNG7	0	0	7	2	0	1	0	0	0	0	0	0	3	0	0	2	0	0
d__Bacteria;k__norank;p__Proteobacteria;c__Alphaproteobacteria;o__Rhizobiales;f__Phyllobacteriaceae;g__Mesorhizobium	22	3	12	4	1	7	4	1	0	9	5	3	20	5	2	5	3	13
d__Bacteria;k__norank;p__Proteobacteria;c__Alphaproteobacteria;o__Rhizobiales;f__Rhizobiaceae;g__Rhizobium	117	143	116	33	24	33	46	48	23	59	197	69	44	50	64	38	57	24
d__Bacteria;k__norank;p__Proteobacteria;c__Alphaproteobacteria;o__Rhizobiales;f__Rhizobiales_Incertae_Sedis;g__Rhizomicrobium	2	8	0	1	0	3	0	0	0	0	0	0	0	0	0	8	0	0
d__Bacteria;k__norank;p__Proteobacteria;c__Alphaproteobacteria;o__Rhizobiales;f__unclassified_o__Rhizobiales;g__unclassified_o__Rhizobiales	0	5	4	3	0	0	0	0	0	0	5	11	0	1	0	0	0	1
d__Bacteria;k__norank;p__Proteobacteria;c__Alphaproteobacteria;o__Rhizobiales;f__Xanthobacteraceae;g__Pseudolabrys	0	0	0	0	4	0	0	0	0	5	11	0	0	0	0	0	0	0
d__Bacteria;k__norank;p__Proteobacteria;c__Alphaproteobacteria;o__Rhizobiales;f__Xanthobacteraceae;g__unclassified_f__Xanthobacteraceae	10	9	0	3	1	0	4	1	0	1	0	0	4	7	0	0	0	0
d__Bacteria;k__norank;p__Proteobacteria;c__Alphaproteobacteria;o__Rhodobacterales;f__Rhodobacteraceae;g__Paracoccus	7	19	10	5	0	0	0	1	1	0	6	14	10	2	9	0	0	16
d__Bacteria;k__norank;p__Proteobacteria;c__Alphaproteobacteria;o__Rhodobacterales;f__Rhodobacteraceae;g__Rhodobacter	0	0	8	0	0	0	0	6	0	0	0	0	4	0	0	0	1	0
d__Bacteria;k__norank;p__Proteobacteria;c__Alphaproteobacteria;o__Rhodobacterales;f__Rhodobacteraceae;g__Rubellimicrobium	8	0	2	0	0	0	1	0	2	0	5	0	0	1	0	1	0	0
d__Bacteria;k__norank;p__Proteobacteria;c__Alphaproteobacteria;o__Rhodobacterales;f__Rhodobacteraceae;g__Rubribacterium	0	0	0	0	0	0	0	0	0	0	0	0	9	0	0	0	0	0

d__Bacteria;k__norank;p__Proteobacteria;c__Alphaproteobacteria;o__Rhodobacterales;f__Rhodobacteraceae;g__unclassified_f__Rhodobacteraceae	0	2	0	0	1	0	0	0	3	0	0	0	7	0	0	3	0	0
d__Bacteria;k__norank;p__Proteobacteria;c__Alphaproteobacteria;o__Rhodospirillales;f__Acetobacteraceae;g__Acidisoma	0	0	0	0	0	0	0	0	1	0	0	4	0	0	0	0	0	0
d__Bacteria;k__norank;p__Proteobacteria;c__Alphaproteobacteria;o__Rhodospirillales;f__Acetobacteraceae;g__Acidocella	13	10	16	4	4	1	6	3	0	9	16	6	4	6	4	4	1	0
d__Bacteria;k__norank;p__Proteobacteria;c__Alphaproteobacteria;o__Rhodospirillales;f__B79;g__norank_f__B79	37	118	82	20	25	12	19	17	13	23	61	19	13	31	30	19	19	7
d__Bacteria;k__norank;p__Proteobacteria;c__Alphaproteobacteria;o__Rhodospirillales;f__I-10;g__norank_f__I-10	0	0	1	2	0	1	1	0	0	8	0	0	1	0	1	3	1	0
d__Bacteria;k__norank;p__Proteobacteria;c__Alphaproteobacteria;o__Rhodospirillales;f__MSB-1E8;g__norank_f__MSB-1E8	13	0	0	0	0	0	1	2	1	0	1	2	6	1	0	3	2	0
d__Bacteria;k__norank;p__Proteobacteria;c__Alphaproteobacteria;o__Rhodospirillales;f__Rhodospirillaceae;g__Inquilinus	0	11	0	0	0	0	0	0	0	0	0	0	0	0	0	0	0	0
d__Bacteria;k__norank;p__Proteobacteria;c__Alphaproteobacteria;o__Rhodospirillales;f__Rhodospirillaceae;g__norank_f__Rhodospirillaceae	0	23	1	0	0	1	0	0	0	2	1	1	0	1	3	0	1	0
d__Bacteria;k__norank;p__Proteobacteria;c__Alphaproteobacteria;o__Rhodospirillales;f__Rhodospirillaceae;g__Skermanella	9	0	0	0	0	0	1	0	0	0	0	0	3	0	21	0	0	0
d__Bacteria;k__norank;p__Proteobacteria;c__Alphaproteobacteria;o__Rhodospirillales;f__Rhodospirillales_Incertae_Sedis;g__Reyranella	8	19	6	4	7	4	2	1	0	4	7	13	0	6	1	0	5	0
d__Bacteria;k__norank;p__Proteobacteria;c__Alphaproteobacteria;o__Rickettsiales;f__Anaplasmataceae;g__Neorickettsia	0	0	0	0	0	0	0	0	0	0	0	0	20	0	0	0	0	0
d__Bacteria;k__norank;p__Proteobacteria;c__Alphaproteobacteria;o__Rickettsiales;f__Holosporaceae;g__norank_f__Holosporaceae	0	8	0	0	0	0	0	0	0	1	0	0	0	0	0	0	0	15

d__Bacteria;k__norank;p__Proteobacteria;c__Alphaproteobacteria;o__Rickettsiales;f__Mitochondria;g__norank_f__Mitochondria	2100	1572	748	587	530	330	346	859	1008	1329	462	1852	4046	556	2757	1074	808	758
d__Bacteria;k__norank;p__Proteobacteria;c__Alphaproteobacteria;o__Rickettsiales;f__TK34;g__norank_f__TK34	0	11	4	2	0	1	1	0	1	0	11	0	0	0	0	1	1	0
d__Bacteria;k__norank;p__Proteobacteria;c__Alphaproteobacteria;o__Sphingomonadales;f__Sphingomonadaceae;g__Sandaracinobacter	0	0	2	0	0	0	0	0	0	0	0	0	0	0	0	1	0	21
d__Bacteria;k__norank;p__Proteobacteria;c__Alphaproteobacteria;o__Sphingomonadales;f__Sphingomonadaceae;g__Sphingobium	184	195	274	53	66	84	63	78	33	150	273	148	56	57	121	112	72	60
d__Bacteria;k__norank;p__Proteobacteria;c__Alphaproteobacteria;o__Sphingomonadales;f__Sphingomonadaceae;g__Sphingomonas	72	54	94	15	19	15	25	25	11	31	95	31	27	37	14	12	23	35
d__Bacteria;k__norank;p__Proteobacteria;c__Alphaproteobacteria;o__Sphingomonadales;f__Sphingomonadaceae;g__unclassified_f__Sphingomonadaceae	2	0	0	0	0	0	0	0	0	0	0	2	2	0	0	0	0	0
d__Bacteria;k__norank;p__Proteobacteria;c__Betaproteobacteria;o__Burkholderiales;f__Alcaligenaceae;g__Achromobacter	11	25	32	4	7	4	4	3	7	6	32	34	7	8	16	23	15	14
d__Bacteria;k__norank;p__Proteobacteria;c__Betaproteobacteria;o__Burkholderiales;f__Burkholderiaceae;g__Burkholderia-Paraburkholderia	1046	1303	1680	318	362	398	519	477	182	688	1406	589	408	478	435	373	432	310
d__Bacteria;k__norank;p__Proteobacteria;c__Betaproteobacteria;o__Burkholderiales;f__Burkholderiaceae;g__Ralstonia	3552	3833	5169	1007	560	820	1240	977	688	876	1681	1950	1444	2001	1752	1325	803	629
d__Bacteria;k__norank;p__Proteobacteria;c__Betaproteobacteria;o__Burkholderiales;f__Comamonadaceae;g__Acidovorax	471	600	629	132	138	166	257	243	102	311	738	279	159	138	189	191	160	184
d__Bacteria;k__norank;p__Proteobacteria;c__Betaproteobacteria;o__Burkholderiales;f__Comamonadaceae;g__Aquabacterium	9	4	17	1	3	2	1	2	1	1	19	9	2	4	1	13	2	0

d__Bacteria;k__norank;p__Proteobacteria;c__Betaproteobacteria;o__Burkholderiales;f__Comamonadaceae;g__Delftia	14	35	33	8	3	12	12	7	4	20	46	12	15	5	2	9	12	12
d__Bacteria;k__norank;p__Proteobacteria;c__Betaproteobacteria;o__Burkholderiales;f__Comamonadaceae;g__Limnohabitans	0	6	0	0	0	0	0	0	0	0	0	0	0	0	0	0	0	0
d__Bacteria;k__norank;p__Proteobacteria;c__Betaproteobacteria;o__Burkholderiales;f__Comamonadaceae;g__Pelomonas	15	14	24	4	7	7	4	0	3	8	9	12	6	4	12	1	3	1
d__Bacteria;k__norank;p__Proteobacteria;c__Betaproteobacteria;o__Burkholderiales;f__Comamonadaceae;g__unclassified_f__Comamonadaceae	7	0	4	0	1	1	0	3	1	0	6	0	1	0	0	0	1	1
d__Bacteria;k__norank;p__Proteobacteria;c__Betaproteobacteria;o__Burkholderiales;f__Oxalobacteraceae;g__Massilia	10	1	0	0	0	0	1	1	0	0	3	3	2	3	3	2	0	0
d__Bacteria;k__norank;p__Proteobacteria;c__Betaproteobacteria;o__Burkholderiales;f__Oxalobacteraceae;g__unclassified_f__Oxalobacteraceae	2262	2981	3483	648	840	850	1208	1163	425	1652	3749	1270	855	726	880	984	929	906
d__Bacteria;k__norank;p__Proteobacteria;c__Betaproteobacteria;o__Burkholderiales;f__Oxalobacteraceae;g__Undibacterium	0	2	0	0	0	0	0	0	0	0	7	0	0	0	0	0	0	0
d__Bacteria;k__norank;p__Proteobacteria;c__Betaproteobacteria;o__Hydrogenophilales;f__Hydrogenophilaceae;g__Hydrogenophilus	0	0	0	0	0	0	0	0	0	0	13	0	0	0	2	0	0	0
d__Bacteria;k__norank;p__Proteobacteria;c__Betaproteobacteria;o__Neisseriales;f__Neisseriaceae;g__Eikenella	0	0	0	0	0	0	0	1	0	1	12	0	0	0	0	0	0	0
d__Bacteria;k__norank;p__Proteobacteria;c__Betaproteobacteria;o__Neisseriales;f__Neisseriaceae;g__Neisseria	7	2	6	5	9	1	15	0	14	2	91	10	12	12	5	5	25	9
d__Bacteria;k__norank;p__Proteobacteria;c__Betaproteobacteria;o__Neisseriales;f__Neisseriaceae;g__norank_f__Neisseriaceae	6	0	2	0	0	0	1	0	0	0	0	0	0	6	0	0	0	0
d__Bacteria;k__norank;p__Proteobacteria;c__Betaproteobacteria;o__Neisseriales;f__Neisseriaceae;g__unclassified_f__Neisseriaceae	0	0	0	0	2	0	0	2	0	0	0	0	12	17	0	0	0	0

d__Bacteria;k__norank;p__Proteobacteria;c__Betaproteobacteria;o__Nitrosomonadales;f__Nitrosomonadaceae;g__norank_f__Nitrosomonadaceae	8	0	4	0	0	2	0	0	0	0	2	0	0	0	0	0	1	0
d__Bacteria;k__norank;p__Proteobacteria;c__Betaproteobacteria;o__Rhodocyclales;f__Rhodocyclaceae;g__Denitratisoma	11	0	0	1	0	0	0	0	0	0	0	0	0	0	8	0	9	0
d__Bacteria;k__norank;p__Proteobacteria;c__Betaproteobacteria;o__Rhodocyclales;f__Rhodocyclaceae;g__Sulfuritalea	0	2	0	0	0	2	0	0	0	0	0	0	0	2	0	0	0	8
d__Bacteria;k__norank;p__Proteobacteria;c__Betaproteobacteria;o__Rhodocyclales;f__Rhodocyclaceae;g__Thauera	16	17	11	2	5	2	5	2	2	1	5	15	7	4	15	2	9	56
d__Bacteria;k__norank;p__Proteobacteria;c__Betaproteobacteria;o__SC-I-84;f__norank_o__SC-I-84;g__norank_o__SC-I-84	0	0	0	0	0	0	0	0	0	0	0	0	0	0	0	0	0	8
d__Bacteria;k__norank;p__Proteobacteria;c__Betaproteobacteria;o__TRA3-20;f__norank_o__TRA3-20;g__norank_o__TRA3-20	3	0	2	0	0	0	0	0	0	0	0	0	0	0	0	0	0	2
d__Bacteria;k__norank;p__Proteobacteria;c__Deltaproteobacteria;o__Bdellovibrionales;f__Bdellovibrionaceae;g__Bdellovibrio	0	0	0	0	0	0	0	0	0	0	0	0	0	0	0	0	4	0
d__Bacteria;k__norank;p__Proteobacteria;c__Deltaproteobacteria;o__Bdellovibrionales;f__Bdellovibrionaceae;g__OM27_clade	1	0	0	0	0	1	0	0	0	0	0	0	0	0	0	0	1	7
d__Bacteria;k__norank;p__Proteobacteria;c__Deltaproteobacteria;o__Desulfobacterales;f__Desulfobacteraceae;g__Sva0081_sediment_group	0	0	0	0	0	0	0	0	0	0	0	0	0	0	9	0	0	0
d__Bacteria;k__norank;p__Proteobacteria;c__Deltaproteobacteria;o__Desulfovibrionales;f__Desulfovibrionaceae;g__Desulfovibrio	1	17	1	1	1	1	0	0	0	0	0	0	0	0	0	0	0	0
d__Bacteria;k__norank;p__Proteobacteria;c__Deltaproteobacteria;o__Desulfurellales;f__Desulfurellaceae;g__H16	0	0	2	2	0	0	0	0	0	0	0	0	0	0	0	0	3	0
d__Bacteria;k__norank;p__Proteobacteria;c__Deltaproteobacteria;o__Myxococcales;f__BIrii41;g__norank_f__BIrii41	0	0	3	0	0	0	0	0	0	0	0	0	0	0	0	0	2	40

d__Bacteria;k__norank;p__Proteobacteria;c__Deltaproteobacteria;o__Myxococcales;f__Haliangiaceae;g__Haliangium	0	7	0	0	0	0	0	0	0	0	0	0	0	0	0	0	0	0
d__Bacteria;k__norank;p__Proteobacteria;c__Deltaproteobacteria;o__Myxococcales;f__mle1-27;g__norank_f__mle1-27	1	10	1	1	0	3	0	0	0	0	0	3	4	2	7	0	1	8
d__Bacteria;k__norank;p__Proteobacteria;c__Deltaproteobacteria;o__Myxococcales;f__Polyangiaceae;g__Sorangium	0	0	3	0	0	0	1	1	0	0	6	3	0	2	0	0	0	0
d__Bacteria;k__norank;p__Proteobacteria;c__Deltaproteobacteria;o__Oligoflexales;f__0319-6G20;g__norank_f__0319-6G20	12	2	12	3	3	2	2	2	0	2	13	1	2	14	4	7	4	5
d__Bacteria;k__norank;p__Proteobacteria;c__Deltaproteobacteria;o__Oligoflexales;f__Oligoflexaceae;g__norank_f__Oligoflexaceae	0	0	0	0	0	0	0	0	0	0	0	0	0	24	0	0	0	0
d__Bacteria;k__norank;p__Proteobacteria;c__Deltaproteobacteria;o__unclassified_c__Deltaproteobacteria;f__unclassified_c__Deltaproteobacteria;g__unclassified_c__Deltaproteobacteria	5	1	4	1	0	5	0	1	0	1	0	6	2	0	2	0	1	7
d__Bacteria;k__norank;p__Proteobacteria;c__Epsilonproteobacteria;o__Campylobacterales;f__Campylobacteraceae;g__Campylobacter	0	2	0	0	0	0	0	1	0	0	0	0	0	0	0	0	1	0
d__Bacteria;k__norank;p__Proteobacteria;c__Epsilonproteobacteria;o__Campylobacterales;f__Helicobacteraceae;g__Helicobacter	3	0	1	1	0	0	1	0	0	0	0	0	0	0	0	0	0	0
d__Bacteria;k__norank;p__Proteobacteria;c__Epsilonproteobacteria;o__Campylobacterales;f__Helicobacteraceae;g__Sulfuricurvum	0	0	0	0	1	0	0	0	0	0	0	0	0	0	0	0	0	11
d__Bacteria;k__norank;p__Proteobacteria;c__Epsilonproteobacteria;o__Campylobacterales;f__Helicobacteraceae;g__Sulfurimonas	0	1	0	0	0	0	0	0	0	0	1	0	2	1	2	0	2	0
d__Bacteria;k__norank;p__Proteobacteria;c__Gammaproteobacteria;o__Aeromonadales;f__Aeromonadaceae;g__Aeromonas	3	0	0	0	0	0	2	1	4	0	0	0	2	12	0	11	0	0

d__Bacteria;k__norank;p__Proteobacteria;c__Gammaproteobacteria;o__Aeromonadales;f__Succinivibrionaceae;g__Anaerobiospirillum	0	0	9	0	0	0	0	0	0	0	0	0	0	0	0	0	0	0
d__Bacteria;k__norank;p__Proteobacteria;c__Gammaproteobacteria;o__Aeromonadales;f__Succinivibrionaceae;g__Succinivibrio	1	5	3	1	1	0	0	1	0	0	0	0	0	0	0	0	0	0
d__Bacteria;k__norank;p__Proteobacteria;c__Gammaproteobacteria;o__Alteromonadales;f__Alteromonadaceae;g__Marinobacter	5	0	0	0	0	0	0	0	0	0	0	0	0	0	0	0	0	0
d__Bacteria;k__norank;p__Proteobacteria;c__Gammaproteobacteria;o__Alteromonadales;f__Shewanellaceae;g__Shewanella	0	0	0	0	0	0	1	4	1	0	0	0	13	89	0	0	0	0
d__Bacteria;k__norank;p__Proteobacteria;c__Gammaproteobacteria;o__Cellvibrionales;f__Cellvibrionaceae;g__Simiduia	0	0	0	0	0	0	0	0	0	0	0	0	0	0	0	0	13	0
d__Bacteria;k__norank;p__Proteobacteria;c__Gammaproteobacteria;o__Enterobacteriales;f__Enterobacteriaceae;g__Escherichia-Shigella	94	141	223	20	33	41	143	126	128	75	153	127	243	180	174	85	103	175
d__Bacteria;k__norank;p__Proteobacteria;c__Gammaproteobacteria;o__Enterobacteriales;f__Enterobacteriaceae;g__Morganella	9	0	0	0	0	0	0	0	0	0	0	0	7	0	0	0	0	0
d__Bacteria;k__norank;p__Proteobacteria;c__Gammaproteobacteria;o__Enterobacteriales;f__Enterobacteriaceae;g__Pantoea	139	188	226	43	54	45	81	78	51	106	230	122	79	79	117	155	90	67
d__Bacteria;k__norank;p__Proteobacteria;c__Gammaproteobacteria;o__Legionellales;f__Legionellaceae;g__Legionella	3	2	4	0	0	0	1	0	0	0	6	4	0	0	1	0	0	0
d__Bacteria;k__norank;p__Proteobacteria;c__Gammaproteobacteria;o__Pasteurellales;f__Pasteurellaceae;g__Aggregatibacter	0	1	0	0	0	3	0	2	0	0	18	0	0	0	0	0	0	5
d__Bacteria;k__norank;p__Proteobacteria;c__Gammaproteobacteria;o__Pasteurellales;f__Pasteurellaceae;g__Haemophilus	0	8	4	3	0	3	1	1	5	1	14	3	0	2	2	2	2	14
d__Bacteria;k__norank;p__Proteobacteria;c__Gammaproteobacteria;o__Pseudomonadales;f__Moraxellaceae;g__Acinetobacter	925	1322	1773	197	486	549	524	1073	256	923	933	551	611	337	432	248	529	543

d__Bacteria;k__norank;p__Proteobacteria;c__Gammaproteobacteria;o__Pseudomonadales;f__Moraxellaceae;g__Enhydrobacter	3	3	6	0	1	1	2	0	0	3	3	3	6	2	0	2	1	10
d__Bacteria;k__norank;p__Proteobacteria;c__Gammaproteobacteria;o__Pseudomonadales;f__Moraxellaceae;g__Moraxella	0	0	5	0	0	1	0	0	2	0	8	0	1	0	0	0	1	2
d__Bacteria;k__norank;p__Proteobacteria;c__Gammaproteobacteria;o__Pseudomonadales;f__Moraxellaceae;g__Perlucidibaca	0	0	9	0	0	0	0	0	0	0	0	0	0	0	0	0	0	0
d__Bacteria;k__norank;p__Proteobacteria;c__Gammaproteobacteria;o__Pseudomonadales;f__Pseudomonadaceae;g__Pseudomonas	463	682	833	167	153	149	255	225	117	295	786	385	206	200	190	160	282	100
d__Bacteria;k__norank;p__Proteobacteria;c__Gammaproteobacteria;o__Run-SP154;f__norank_o__Run-SP154;g__norank_o__Run-SP154	0	0	0	0	0	0	0	0	0	0	0	0	0	0	0	0	0	31
d__Bacteria;k__norank;p__Proteobacteria;c__Gammaproteobacteria;o__unclassified_c__Gammaproteobacteria;f__unclassified_c__Gammaproteobacteria;g__unclassified_c__Gammaproteobacteria	3	3	12	1	1	0	2	0	2	0	6	0	24	0	0	0	0	0
d__Bacteria;k__norank;p__Proteobacteria;c__Gammaproteobacteria;o__Vibrionales;f__Vibrionaceae;g__Photobacterium	0	0	0	0	6	0	0	3	0	0	0	0	5	3541	0	0	18	0
d__Bacteria;k__norank;p__Proteobacteria;c__Gammaproteobacteria;o__Vibrionales;f__Vibrionaceae;g__Vibrio	7	0	0	0	4	0	0	4	0	0	0	0	16	2689	1	0	12	10
d__Bacteria;k__norank;p__Proteobacteria;c__Gammaproteobacteria;o__Xanthomonadales;f__JTB255_marine_benthic_group;g__norank_f__JTB255_marine_benthic_group	0	0	9	0	0	0	0	0	0	0	0	0	0	0	0	0	0	0
d__Bacteria;k__norank;p__Proteobacteria;c__Gammaproteobacteria;o__Xanthomonadales;f__norank_o__Xanthomonadales;g__norank_o__Xanthomonadales	0	4	2	0	0	1	0	0	0	0	1	0	0	0	0	0	0	0

d__Bacteria;k__norank;p__Proteobacteria;c__Gammaproteobacteria;o__Xanthomonadales;f__Xanthomonadaceae;g__Dokdonella	6	0	0	0	0	0	0	0	0	1	0	0	3	0	0	0	0	0
d__Bacteria;k__norank;p__Proteobacteria;c__Gammaproteobacteria;o__Xanthomonadales;f__Xanthomonadaceae;g__Dyella	5	6	7	0	1	1	5	1	1	3	2	3	0	0	3	1	0	0
d__Bacteria;k__norank;p__Proteobacteria;c__Gammaproteobacteria;o__Xanthomonadales;f__Xanthomonadaceae;g__Ignatzschineria	0	0	0	0	0	0	0	0	0	0	0	9	0	0	0	0	0	0
d__Bacteria;k__norank;p__Proteobacteria;c__Gammaproteobacteria;o__Xanthomonadales;f__Xanthomonadaceae;g__norank_f__Xanthomonadaceae	5	1	4	0	0	0	1	0	0	0	0	0	0	0	0	0	0	0
d__Bacteria;k__norank;p__Proteobacteria;c__Gammaproteobacteria;o__Xanthomonadales;f__Xanthomonadaceae;g__Stenotrophomonas	144	141	233	31	40	65	65	45	35	69	167	58	49	66	73	56	30	47
d__Bacteria;k__norank;p__Proteobacteria;c__Gammaproteobacteria;o__Xanthomonadales;f__Xanthomonadaceae;g__Tahibacter	0	0	0	0	0	0	0	1	1	0	0	0	3	0	0	0	0	0
d__Bacteria;k__norank;p__Proteobacteria;c__Gammaproteobacteria;o__Xanthomonadales;f__Xanthomonadaceae;g__Thermomonas	22	31	27	1	9	7	4	4	3	17	27	8	8	3	7	2	2	12
d__Bacteria;k__norank;p__Proteobacteria;c__Gammaproteobacteria;o__Xanthomonadales;f__Xanthomonadaceae;g__unclassified_f__Xanthomonadaceae	0	13	0	2	0	0	0	0	0	0	0	1	0	0	0	0	0	0
d__Bacteria;k__norank;p__Proteobacteria;c__Proteobacteria_Incertae_Sedis;o__Unknown_c__Proteobacteria_Incertae_Sedis;f__Unknown_o__Unknown_c__Proteobacteria_Incertae_Sedis;g__Candidatus_Thiobios	0	0	0	0	0	0	0	0	0	0	0	0	13	0	0	0	0	0
d__Bacteria;k__norank;p__Saccharibacteria;c__norank_p__Saccharibacteria;o__norank_p__Saccharibacteria;f__norank_p__Saccharibacteria;g__norank_p__Saccharibacteria	1	0	2	1	0	1	2	2	0	3	0	0	1	19	1	0	0	29

d__Bacteria;k__norank;p__Saccharibacteria;c__Unknown_Class_p__Saccharibacteria;o__Unknown_Order_c__Unknown_Class_p__Saccharibacteria;f__Unknown_Family_o__Unknown_Order_c__Unknown_Class_p__Saccharibacteria;g__Candidatus_Saccharimonas	1	12	0	0	0	0	0	0	0	0	0	0	0	0	0	0	0	0
d__Bacteria;k__norank;p__SBR1093;c__norank_p__SBR1093;o__norank_p__SBR1093;f__norank_p__SBR1093;g__norank_p__SBR1093	0	0	0	0	3	0	2	1	0	1	0	3	0	0	0	0	0	0
d__Bacteria;k__norank;p__Spirochaetae;c__Spirochaetes;o__Spirochaetales;f__Spirochaetaceae;g__Sphaerochaeta	0	0	0	1	0	0	0	0	0	0	0	0	0	3	1	0	0	1
d__Bacteria;k__norank;p__Spirochaetae;c__Spirochaetes;o__Spirochaetales;f__Spirochaetaceae;g__Spirochaeta_2	0	3	0	0	0	0	0	0	0	0	0	0	1	0	0	0	0	2
d__Bacteria;k__norank;p__Spirochaetae;c__Spirochaetes;o__Spirochaetales;f__Spirochaetaceae;g__Treponema_2	0	8	0	0	1	2	0	1	2	0	0	0	0	0	3	0	0	0
d__Bacteria;k__norank;p__Synergistetes;c__Synergistia;o__Synergistales;f__Synergistaceae;g__Pyramidobacter	3	0	0	0	0	0	0	0	0	0	0	0	0	0	0	0	0	0
d__Bacteria;k__norank;p__Tenericutes;c__Mollicutes;o__Mollicutes_RF9;f__norank_o__Mollicutes_RF9;g__norank_o__Mollicutes_RF9	0	0	0	0	2	0	0	0	0	0	0	0	0	0	0	0	0	0
d__Bacteria;k__norank;p__Tenericutes;c__Mollicutes;o__Mycoplasmatales;f__Mycoplasmataceae;g__Candidatus_Bacilloplasma	0	0	0	0	0	0	0	5	0	0	0	0	7	29	0	0	3	0
d__Bacteria;k__norank;p__Tenericutes;c__Mollicutes;o__Mycoplasmatales;f__Mycoplasmataceae;g__norank_f__Mycoplasmataceae	0	0	0	0	0	0	0	0	0	0	0	0	0	31	0	0	0	0
*d__Bacteria;k__norank;p__TM6__Dependentiae_;c__norank_p__TM6__Dependentiae_;o__norank_p__TM6__Dependentiae_;f__norank_p__TM6__Dependentiae_;g__norank_p__TM6__Dependentiae_*	3	0	0	0	0	0	0	0	0	0	0	0	0	0	0	0	0	0

d__Bacteria;k__norank;p__unclassified_k__norank;c__unclassified_k__norank;o__unclassified_k__norank;f__unclassified_k__norank;g__unclassified_k__norank	12	10	17	12	14	24	6	7	20	1	8	1	3	3	4	1	4	38
d__Bacteria;k__norank;p__Verrucomicrobia;c__Spartobacteria;o__Chthoniobacterales;f__LD29;g__norank_f__LD29	0	0	0	0	0	0	0	0	0	0	0	0	3	108	0	0	0	0
d__Bacteria;k__norank;p__Verrucomicrobia;c__Verrucomicrobiae;o__Verrucomicrobiales;f__Verrucomicrobiaceae;g__Akkermansia	1	2	3	0	0	5	0	1	1	0	0	0	6	0	2	0	0	0
d__Bacteria;k__norank;p__WS6;c__norank_p__WS6;o__norank_p__WS6;f__norank_p__WS6;g__norank_p__WS6	0	0	0	0	0	0	0	0	0	0	0	0	0	9	0	0	8	0
d__Bacteria;k__norank;p__WWE3;c__norank_p__WWE3;o__norank_p__WWE3;f__norank_p__WWE3;g__norank_p__WWE3	0	0	0	0	0	0	0	0	0	0	0	0	0	18	0	0	0	0

Table S4. Correlation between HA and microorganisms on genus level.

HA	Microorganisms	P value	P adjust	correlation
1-Dodecanol	*Anaerotruncus*	0.050	0.566	(0.812)
	Candidatus_Thiobios	0.044	0.520	0.824
	CL500-29_marine_group	0.044	0.520	0.824
	CL500-3	0.044	0.520	0.824
	Clostridium_sensu_stricto_13	0.030	0.453	0.854
	Deinococcus	0.019	0.340	0.884
	Ezakiella	0.044	0.520	0.824
	Fastidiosipila	0.044	0.520	0.824
	Flectobacillus	0.044	0.520	0.824
	Gallicola	0.044	0.520	0.824
	hgcI_clade	0.044	0.520	0.824
	Hirschia	0.044	0.520	0.824
	Ilumatobacter	0.022	0.377	0.875
	Kocuria	0.034	0.474	0.846
	Lachnoclostridium	0.037	0.490	0.838
	Neorickettsia	0.044	0.520	0.824
	norank_c_vadinHA49	0.044	0.520	0.824
	norank_f_Caldilineaceae	0.045	0.532	0.821
	norank_f_FamilyI	0.020	0.341	0.884
	norank_f_LD29	0.044	0.520	0.824
	norank_f_Leptotrichiaceae	0.044	0.520	0.824
	norank_f_Mycoplasmataceae	0.044	0.520	0.824
	norank_f_Oligoflexaceae	0.044	0.520	0.824
	norank_f_OPB56	0.044	0.520	0.824

	norank_p_Latescibacteria	0.044	0.520	0.824
	norank_p_WWE3	0.044	0.520	0.824
	Pediococcus	0.023	0.386	0.873
	Pedomicrobium	0.039	0.509	0.834
	Peptoniphilus	0.037	0.490	0.838
	Photobacterium	0.043	0.520	0.825
	Propionigenium	0.044	0.520	0.824
	Rubribacterium	0.044	0.520	0.824
	Ruminiclostridium_1	0.044	0.520	0.824
	Sediminibacterium	0.044	0.520	0.824
	Sphaerochaeta	0.044	0.520	0.824
	Spongiimonas	0.044	0.520	0.824
	Sulfurimonas	0.013	0.261	0.905
	Sva0081_sediment_group	0.044	0.520	0.824
	unclassified_f_FamilyI_o_SubsectionI	0.044	0.520	0.824
	unclassified_f_FamilyI_o_SubsectionIII	0.018	0.322	0.889
	unclassified_f_Flavobacteriaceae	0.044	0.520	0.824
	Vibrio	0.043	0.520	0.825
1-Heptadecanol	*[Eubacterium]_nodatum_group*	0.006	0.152	0.937
	Bradyrhizobium	0.017	0.306	(0.893)
	Cellulosimicrobium	0.010	0.212	(0.919)
	Cloacibacterium	0.010	0.218	(0.916)
	Enhydrobacter	0.013	0.260	(0.905)
	Micrococcus	0.038	0.493	(0.837)
	norank_c_Cyanobacteria	0.028	0.438	(0.859)
	norank_f_0319-6G20	0.042	0.520	(0.828)

	Rhodococcus	0.020	0.344	(0.883)
1-Hexacosanol	*[Eubacterium]_hallii_group*	0.004	0.113	0.948
	Abiotrophia	0.000	0.000	0.999
	Acidisoma	0.019	0.333	0.886
	Aggregatibacter	0.012	0.250	0.908
	Bosea	0.015	0.283	0.899
	Capnocytophaga	0.009	0.203	0.922
	Caulobacter	0.010	0.221	0.916
	Eikenella	0.006	0.155	0.936
	Gemella	0.024	0.394	0.871
	Geobacillus	0.000	0.025	0.982
	Hydrogenophilus	0.001	0.031	0.977
	Ignatzschineria	0.004	0.113	0.948
	Neisseria	0.002	0.068	0.963
	norank_f_Erysipelotrichaceae	0.040	0.511	(0.833)
	norank_f_I-10	0.011	0.234	0.912
	Porphyromonas	0.030	0.453	0.854
	Pseudolabrys	0.015	0.288	0.898
	Pseudonocardia	0.004	0.113	0.948
	Sorangium	0.027	0.431	0.862
	unclassified_f_Rikenellaceae	0.003	0.088	0.956
	Undibacterium	0.026	0.412	0.866
	Veillonella	0.006	0.150	0.937
1-Hexadecanol	*Candidatus_Bacilloplasma*	0.041	0.520	0.829
	Candidatus_Thiobios	0.035	0.474	0.843
	CL500-29_marine_group	0.035	0.474	0.843

CL500-3	0.035	0.474	0.843
Deinococcus	0.032	0.472	0.849
Ezakiella	0.035	0.474	0.843
Fastidiosipila	0.035	0.474	0.843
Flectobacillus	0.035	0.474	0.843
Gallicola	0.035	0.474	0.843
hgcI_clade	0.035	0.474	0.843
Hirschia	0.035	0.474	0.843
Ilumatobacter	0.029	0.444	0.858
Kocuria	0.009	0.209	0.920
Lachnoclostridium	0.031	0.461	0.852
Neorickettsia	0.035	0.474	0.843
norank_c_vadinHA49	0.035	0.474	0.843
norank_f_Caldilineaceae	0.036	0.478	0.841
norank_f_FamilyI	0.005	0.128	0.944
norank_f_LD29	0.035	0.474	0.843
norank_f_Leptotrichiaceae	0.035	0.474	0.843
norank_f_Mycoplasmataceae	0.035	0.474	0.843
norank_f_Oligoflexaceae	0.035	0.474	0.843
norank_f_OPB56	0.035	0.474	0.843
norank_o_PeM15	0.043	0.520	0.825
norank_p_Latescibacteria	0.035	0.474	0.843
norank_p_WWE3	0.035	0.474	0.843
Pediococcus	0.014	0.273	0.901
Pedomicrobium	0.014	0.276	0.901
Peptoniphilus	0.029	0.440	0.859

	Photobacterium	0.035	0.474	0.843
	Propionigenium	0.035	0.474	0.843
	Rubribacterium	0.035	0.474	0.843
	Ruminiclostridium_1	0.035	0.474	0.843
	Sediminibacterium	0.035	0.474	0.843
	Shewanella	0.039	0.509	0.833
	Spongiimonas	0.035	0.474	0.843
	Sulfurimonas	0.018	0.329	0.887
	Sva0081_sediment_group	0.035	0.474	0.843
	Synechococcus	0.040	0.517	0.831
	Thermus	0.042	0.520	0.827
	unclassified_f_FamilyI_o_SubsectionI	0.035	0.474	0.843
	unclassified_f_FamilyI_o_SubsectionIII	0.009	0.203	0.922
	unclassified_f_Flavobacteriaceae	0.035	0.474	0.843
	unclassified_f_Neisseriaceae	0.048	0.557	0.815
	unclassified_k_norank	0.041	0.518	(0.830)
	Vibrio	0.035	0.474	0.843
1-Triacontanol	*[Eubacterium]_hallii_group*	0.005	0.131	0.942
	Abiotrophia	0.000	0.007	0.992
	Acidisoma	0.019	0.339	0.884
	Aggregatibacter	0.023	0.378	0.874
	Bosea	0.010	0.214	0.918
	Capnocytophaga	0.011	0.227	0.914
	Caulobacter	0.007	0.176	0.930
	Cetobacterium	0.035	0.474	0.842
	Eikenella	0.007	0.175	0.931

	Gemella	0.023	0.387	0.873
	Geobacillus	0.001	0.032	0.977
	Hydrogenophilus	0.001	0.025	0.981
	Ignatzschineria	0.005	0.131	0.942
	Neisseria	0.005	0.133	0.942
	norank_f_I-10	0.025	0.404	0.868
	Porphyromonas	0.033	0.474	0.847
	Pseudolabrys	0.018	0.322	0.888
	Pseudonocardia	0.005	0.131	0.942
	Sorangium	0.019	0.331	0.886
	unclassified_f_Rikenellaceae	0.001	0.025	0.981
	Undibacterium	0.024	0.397	0.870
	Veillonella	0.010	0.217	0.917
	Vibrionimonas	0.043	0.520	0.826
1-Undecanol	*norank_o_JG30-KF-CM45*	0.001	0.036	0.975
	Truepera	0.000	0.001	0.997
2-(2-butoxyethoxy)-Ethanol	*[Eubacterium]_eligens_group*	0.042	0.520	(0.827)
	Anaerococcus	0.029	0.444	0.858
	Anaerotruncus	0.029	0.448	(0.857)
	Filifactor	0.035	0.474	0.843
	Finegoldia	0.019	0.338	0.885
	Lachnospiraceae_NK4A136_group	0.049	0.562	(0.813)
	norank_p_Saccharibacteria	0.009	0.209	0.920
	norank_p_WS6	0.028	0.433	0.861
	Prevotella	0.045	0.525	0.822
2-ethyl-1-Hexanol	*[Eubacterium]_nodatum_group*	0.047	0.540	0.818

	Achromobacter	0.042	0.520	(0.827)
	Bradyrhizobium	0.008	0.188	(0.926)
	Cellulosimicrobium	0.026	0.420	(0.865)
	Cloacibacterium	0.004	0.118	(0.947)
	Enhydrobacter	0.009	0.209	(0.920)
	Micrococcus	0.032	0.463	(0.851)
	norank_c_Cyanobacteria	0.045	0.532	(0.821)
	norank_f_0319-6G20	0.020	0.344	(0.882)
	Paracoccus	0.040	0.510	(0.833)
	Rhodococcus	0.017	0.310	(0.892)
2-ethyl-2-methyl-Tridecanol	*Anaerotruncus*	0.034	0.474	(0.847)
	Bradyrhizobium	0.037	0.493	0.837
	Cloacibacterium	0.046	0.534	0.820
	Clostridium_sensu_stricto_13	0.007	0.175	0.931
	Deinococcus	0.010	0.218	0.917
	Granulicatella	0.027	0.425	0.863
	Ilumatobacter	0.022	0.365	0.878
	norank_p_Saccharibacteria	0.047	0.546	0.817
	norank_p_WS6	0.012	0.237	0.911
	Sulfurimonas	0.009	0.209	0.920
2-Furanmethanol	*[Eubacterium]_coprostanoligenes_group*	0.001	0.048	0.970
	Acinetobacter	0.013	0.257	0.906
	Alistipes	0.000	0.000	0.998
	Alloprevotella	0.001	0.028	0.978
	Anaerobiospirillum	0.000	0.000	0.999
	Anaerostipes	0.010	0.218	0.916

Aspergillus	0.000	0.005	0.993
Bacillus	0.002	0.071	0.962
Blautia	0.010	0.217	0.917
Burkholderia-Paraburkholderia	0.030	0.453	0.855
Candidatus_Saccharimonas	0.000	0.000	0.999
Catenibacterium	0.000	0.021	0.984
Christensenellaceae_R-7_group	0.007	0.176	0.929
Chroococcidiopsis	0.016	0.294	0.896
Clostridium_sensu_stricto_15	0.001	0.037	0.975
Coprococcus_3	0.004	0.113	0.949
Desulfovibrio	0.000	0.007	0.991
Devosia	0.000	0.000	0.999
Dialister	0.002	0.056	0.967
Dokdonella	0.025	0.407	0.867
Dyella	0.016	0.300	0.894
Enorma	0.000	0.000	0.999
Enterococcus	0.000	0.010	0.990
Erysipelotrichaceae_UCG-002	0.027	0.423	0.864
Faecalibacterium	0.000	0.016	0.986
Faecalibaculum	0.031	0.453	0.854
Family_XIII_AD3011_group	0.004	0.118	0.947
Haliangium	0.000	0.000	0.999
Helicobacter	0.003	0.080	0.958
Holdemanella	0.002	0.053	0.968
Inquilinus	0.000	0.000	0.999
Kroppenstedtia	0.007	0.176	0.930

Lachnospiraceae_UCG-010	0.000	0.000	0.999
Lactococcus	0.041	0.518	(0.831)
Lawsonella	0.001	0.048	0.970
Limnohabitans	0.000	0.000	0.999
Marinobacter	0.000	0.000	0.999
Marvinbryantia	0.015	0.286	0.898
Megasphaera	0.015	0.286	0.898
Microlunatus	0.012	0.242	0.910
Mycobacterium	0.012	0.248	0.909
Nakamurella	0.001	0.045	0.972
Nitrolancea	0.001	0.048	0.970
Nitrospira	0.014	0.267	0.903
norank_c_Acidobacteria	0.003	0.093	0.955
norank_c_Bacteroidetes_BD2-2	0.000	0.000	0.999
norank_c_Bacteroidetes_VC2.1_Bac22	0.000	0.000	0.999
norank_c_BD7-11	0.001	0.033	0.977
norank_f_Acidimicrobiaceae	0.000	0.000	0.999
norank_f_Acidobacteriaceae_Subgroup_1	0.000	0.000	0.999
norank_f_Actinopolysporaceae	0.026	0.415	0.866
norank_f_B79	0.003	0.100	0.952
norank_f_Bacteroidales_BS11_gut_group	0.008	0.179	0.928
norank_f_Bacteroidales_S24-7_group	0.002	0.065	0.964
norank_f_Bacteroidales_UCG-001	0.024	0.392	0.871
norank_f_Chitinophagaceae	0.000	0.004	0.994
norank_f_Clostridiales_vadinBB60_group	0.000	0.000	0.999
norank_f_Gemmatimonadaceae	0.000	0.000	0.999

norank_f_JTB255_marine_benthic_group	0.000	0.000	0.999
norank_f_Lachnospiraceae	0.017	0.317	0.890
norank_f_MNG7	0.030	0.453	0.854
norank_f_MSB-1E8	0.038	0.495	0.837
norank_f_Nitrosomonadaceae	0.001	0.025	0.982
norank_f_Rhodospirillaceae	0.001	0.028	0.979
norank_f_Xanthomonadaceae	0.000	0.003	0.996
norank_f_XIII	0.000	0.000	0.999
norank_o_AKYG1722	0.000	0.000	0.999
norank_o_Gaiellales	0.030	0.453	0.854
norank_o_Limnochordales	0.000	0.000	0.999
norank_o_Obscuribacterales	0.000	0.013	0.988
norank_o_Subgroup_7	0.000	0.000	0.999
norank_o_TRA3-20	0.011	0.226	0.914
norank_o_Xanthomonadales	0.001	0.025	0.980
norank_p_Parcubacteria	0.013	0.260	0.905
norank_p_TM6_Dependentiae	0.000	0.000	0.999
Oceanobacillus	0.010	0.212	0.919
Odoribacter	0.012	0.248	0.908
Oribacterium	0.003	0.080	0.958
Oscillibacter	0.006	0.151	0.937
Paenibacillus	0.034	0.474	0.846
Papillibacter	0.000	0.000	0.999
Parabacteroides	0.000	0.005	0.993
Pelomonas	0.031	0.459	0.852
Perlucidibaca	0.000	0.000	0.999

Phascolarctobacterium	0.021	0.356	0.880
Prevotella_1	0.001	0.032	0.977
Prevotella_9	0.007	0.175	0.930
Prevotellaceae_NK3B31_group	0.001	0.045	0.972
Prevotellaceae_UCG-001	0.001	0.025	0.981
Prevotellaceae_UCG-003	0.000	0.002	0.997
Pseudoclavibacter	0.005	0.129	0.943
Pyramidobacter	0.000	0.000	0.999
Ralstonia	0.004	0.106	0.951
Rikenellaceae_RC9_gut_group	0.002	0.065	0.964
Rubellimicrobium	0.024	0.395	0.870
Ruminococcaceae_UCG-002	0.010	0.214	0.918
Ruminococcaceae_UCG-007	0.000	0.000	0.999
Ruminococcaceae_UCG-010	0.000	0.000	0.999
Ruminococcaceae_UCG-011	0.007	0.176	0.929
Ruminococcus_1	0.000	0.001	0.998
Ruminococcus_2	0.002	0.072	0.961
Saccharomyces	0.014	0.278	(0.900)
Saccharopolyspora	0.002	0.068	0.963
Sedimentibacter	0.000	0.000	0.999
Selenomonas	0.000	0.004	0.994
Sphingomonas	0.049	0.561	0.814
Stenotrophomonas	0.012	0.248	0.908
Streptococcus	0.016	0.300	0.894
Streptomyces	0.029	0.444	0.858
Subdoligranulum	0.000	0.020	0.984

	Succinivibrio	0.001	0.025	0.981
	Thalassobacillus	0.000	0.000	0.999
	Thermomonas	0.038	0.495	0.837
	Treponema_2	0.022	0.377	0.875
	Tyzzerella_3	0.000	0.000	0.999
	unclassified_f_Comamonadaceae	0.023	0.378	0.875
	unclassified_f_Coriobacteriaceae	0.000	0.000	0.999
	unclassified_f_Microbacteriaceae	0.000	0.000	0.999
	unclassified_f_Nocardioidaceae	0.000	0.000	0.999
	unclassified_f_Prevotellaceae	0.000	0.007	0.991
	unclassified_f_Xanthobacteraceae	0.035	0.474	0.842
	unclassified_f_Xanthomonadaceae	0.000	0.008	0.991
	unclassified_o_Chlamydiales	0.000	0.000	0.999
	unclassified_o_Solirubrobacterales	0.000	0.000	0.999
2-Isopropyl-5-methyl-1-heptanol	*Bradyrhizobium*	0.025	0.401	(0.869)
	Cloacibacterium	0.020	0.341	(0.883)
	Enhydrobacter	0.037	0.492	(0.838)
	norank_f_0319-6G20	0.028	0.433	(0.861)
	norank_o_JG30-KF-CM45	0.032	0.463	0.851
	Truepera	0.007	0.177	0.929
2-methyl-1-Propanol	*Acidothermus*	0.000	0.000	1.000
	Anaerococcus	0.000	0.020	0.984
	Bdellovibrio	0.000	0.000	1.000
	Brevibacterium	0.000	0.000	0.999
	Candidatus_Kuenenia	0.000	0.004	0.994
	Filifactor	0.023	0.378	0.875

Finegoldia	0.005	0.127	0.944
Lachnoanaerobaculum	0.002	0.056	0.967
Lactobacillus	0.018	0.318	(0.890)
norank_c_SJA-15	0.003	0.093	0.955
norank_f_Anaerolineaceae	0.006	0.160	0.935
norank_f_BIrii41	0.000	0.002	0.996
norank_f_Holosporaceae	0.029	0.440	0.859
norank_f_NS9_marine_group	0.000	0.000	1.000
norank_o_Acidimicrobiales	0.000	0.003	0.996
norank_o_Run-SP154	0.000	0.000	1.000
norank_o_SC-I-84	0.000	0.000	1.000
OM27_clade	0.000	0.020	0.984
Paludibaculum	0.030	0.448	0.856
Podosphaera	0.005	0.142	0.939
Prevotella	0.018	0.322	0.889
Propioniciclava	0.000	0.000	1.000
Rothia	0.001	0.025	0.980
Sandaracinobacter	0.000	0.004	0.994
Scardovia	0.000	0.000	1.000
Schwartzia	0.000	0.000	1.000
Simiduia	0.000	0.000	1.000
Sphingobacterium	0.000	0.000	1.000
Sulfuricurvum	0.000	0.004	0.995
Sulfuritalea	0.005	0.131	0.943
Thauera	0.046	0.539	0.819
Virgibacillus	0.045	0.527	0.822

	Woodsholea	0.000	0.000	1.000
2-O-decyl-Threitol	*[Eubacterium]_coprostanoligenes_group*	0.006	0.144	0.939
	Acinetobacter	0.019	0.337	0.885
	Alistipes	0.002	0.058	0.966
	Alloprevotella	0.009	0.203	0.922
	Anaerobiospirillum	0.001	0.025	0.980
	Anaerostipes	0.040	0.512	0.832
	Aspergillus	0.001	0.030	0.978
	Bacillus	0.000	0.025	0.982
	Blautia	0.002	0.073	0.961
	Brevundimonas	0.035	0.474	0.842
	Burkholderia-Paraburkholderia	0.028	0.434	0.860
	Candidatus_Saccharimonas	0.001	0.025	0.980
	Catenibacterium	0.010	0.212	0.919
	Christensenellaceae_R-7_group	0.007	0.176	0.929
	Chroococcidiopsis	0.018	0.318	0.889
	Clostridium_sensu_stricto_15	0.010	0.218	0.916
	Coprococcus_3	0.031	0.459	0.852
	Desulfovibrio	0.004	0.118	0.947
	Devosia	0.001	0.046	0.971
	Dialister	0.006	0.164	0.933
	Dokdonella	0.009	0.203	0.922
	Dyella	0.031	0.453	0.854
	Enorma	0.001	0.025	0.980
	Enterococcus	0.004	0.112	0.949
	Erysipelotrichaceae_UCG-002	0.042	0.520	0.827

Faecalibacterium	0.004	0.118	0.947
Faecalibaculum	0.031	0.453	0.854
Family_XIII_AD3011_group	0.021	0.359	0.879
Haliangium	0.001	0.025	0.980
Helicobacter	0.024	0.394	0.871
Holdemanella	0.013	0.264	0.904
Inquilinus	0.001	0.025	0.980
Kroppenstedtia	0.003	0.085	0.957
Lachnospiraceae_UCG-010	0.001	0.025	0.980
Lactococcus	0.007	0.176	(0.929)
Lawsonella	0.000	0.016	0.986
Limnohabitans	0.001	0.025	0.980
Marinobacter	0.001	0.025	0.980
Marvinbryantia	0.008	0.191	0.925
Megasphaera	0.009	0.210	0.919
Mesorhizobium	0.031	0.461	0.852
Microlunatus	0.008	0.190	0.925
Mycobacterium	0.002	0.070	0.962
Nakamurella	0.002	0.059	0.966
Nitrolancea	0.008	0.196	0.924
Nitrospira	0.016	0.300	0.894
norank_c_Acidobacteria	0.005	0.131	0.943
norank_c_Bacteroidetes_BD2-2	0.001	0.025	0.980
norank_c_Bacteroidetes_VC2.1_Bac22	0.001	0.025	0.980
norank_c_BD7-11	0.001	0.053	0.969
norank_c_Cyanobacteria	0.047	0.540	0.818

norank_f_Acidimicrobiaceae	0.001	0.025	0.980
norank_f_Acidobacteriaceae_Subgroup_1	0.001	0.025	0.980
norank_f_Actinopolysporaceae	0.018	0.327	0.887
norank_f_B79	0.004	0.106	0.951
norank_f_Bacteroidales_BS11_gut_group	0.012	0.237	0.911
norank_f_Bacteroidales_S24-7_group	0.006	0.163	0.934
norank_f_Bacteroidales_UCG-001	0.033	0.473	0.849
norank_f_Chitinophagaceae	0.001	0.048	0.970
norank_f_Clostridiales_vadinBB60_group	0.001	0.025	0.980
norank_f_Gemmatimonadaceae	0.001	0.025	0.980
norank_f_JTB255_marine_benthic_group	0.001	0.025	0.980
norank_f_Lachnospiraceae	0.014	0.272	0.902
norank_f_MNG7	0.028	0.434	0.860
norank_f_MSB-1E8	0.009	0.203	0.922
norank_f_Neisseriaceae	0.049	0.562	0.813
norank_f_Nitrosomonadaceae	0.004	0.113	0.948
norank_f_Rhodospirillaceae	0.000	0.018	0.985
norank_f_Xanthomonadaceae	0.002	0.061	0.965
norank_f_XIII	0.001	0.025	0.980
norank_o_AKYG1722	0.001	0.025	0.980
norank_o_Gaiellales	0.016	0.294	0.896
norank_o_Limnochordales	0.001	0.025	0.980
norank_o_Obscuribacterales	0.001	0.039	0.974
norank_o_Subgroup_7	0.001	0.025	0.980
norank_o_TRA3-20	0.006	0.150	0.937
norank_o_Xanthomonadales	0.006	0.147	0.938

norank_p_Parcubacteria	0.016	0.300	0.894
*norank_p_TM6_Dependentiae_*	0.001	0.025	0.980
Oceanobacillus	0.020	0.344	0.882
Oribacterium	0.024	0.394	0.871
Oscillibacter	0.029	0.447	0.857
Paenibacillus	0.039	0.504	0.835
Papillibacter	0.001	0.025	0.980
Parabacteroides	0.001	0.047	0.971
Paracoccus	0.025	0.404	0.868
Pelomonas	0.032	0.463	0.851
Perlucidibaca	0.001	0.025	0.980
Phascolarctobacterium	0.020	0.342	0.883
Prevotella_1	0.012	0.237	0.911
Prevotella_7	0.044	0.525	0.823
Prevotella_9	0.039	0.510	0.833
Prevotellaceae_NK3B31_group	0.011	0.229	0.913
Prevotellaceae_UCG-001	0.002	0.064	0.964
Prevotellaceae_UCG-003	0.003	0.096	0.954
Pseudoclavibacter	0.001	0.045	0.971
Pyramidobacter	0.001	0.025	0.980
Ralstonia	0.001	0.034	0.976
Rikenellaceae_RC9_gut_group	0.004	0.120	0.946
Rubellimicrobium	0.029	0.445	0.857
Ruminococcaceae_UCG-002	0.044	0.520	0.824
Ruminococcaceae_UCG-007	0.001	0.025	0.980
Ruminococcaceae_UCG-010	0.001	0.025	0.980

Ruminococcaceae_UCG-011	0.007	0.175	0.931
Ruminococcus_1	0.001	0.035	0.975
Ruminococcus_2	0.009	0.209	0.920
Saccharomyces	0.016	0.294	(0.896)
Saccharopolyspora	0.003	0.082	0.958
Sedimentibacter	0.001	0.025	0.980
Selenomonas	0.002	0.073	0.961
Sphingomonas	0.035	0.474	0.842
Spirochaeta_2	0.030	0.450	0.856
Stenotrophomonas	0.010	0.218	0.916
Streptococcus	0.007	0.177	0.929
Streptomyces	0.009	0.207	0.921
Subdoligranulum	0.008	0.188	0.926
Succinivibrio	0.011	0.228	0.914
Thalassobacillus	0.001	0.025	0.980
Thermomonas	0.035	0.474	0.843
Tyzzerella_3	0.001	0.025	0.980
unclassified_f_Comamonadaceae	0.041	0.518	0.830
unclassified_f_Coriobacteriaceae	0.001	0.025	0.980
unclassified_f_Lachnospiraceae	0.041	0.518	0.830
unclassified_f_Microbacteriaceae	0.001	0.025	0.980
unclassified_f_Nocardioidaceae	0.001	0.025	0.980
unclassified_f_Prevotellaceae	0.006	0.148	0.938
unclassified_f_Xanthobacteraceae	0.034	0.474	0.846
unclassified_f_Xanthomonadaceae	0.004	0.113	0.949
unclassified_o_Chlamydiales	0.001	0.025	0.980

	unclassified_o_Solirubrobacterales	0.001	0.025	0.980
2-phenoxy-Ethanol	*Aspergillus*	0.040	0.512	0.832
	Bifidobacterium	0.027	0.424	0.863
	Denitratisoma	0.017	0.306	0.893
	Erysipelotrichaceae_UCG-002	0.023	0.378	0.875
	Lactococcus	0.045	0.526	(0.822)
	Lawsonella	0.044	0.523	0.823
	Lysinibacillus	0.036	0.481	0.840
	Marvinbryantia	0.024	0.396	0.870
	Mesorhizobium	0.030	0.453	0.855
	norank_f_MNG7	0.049	0.561	0.814
	norank_o_Obscuribacterales	0.034	0.474	0.846
	norank_o_TRA3-20	0.003	0.095	0.954
	Parabacteroides	0.037	0.492	0.838
	Prevotella_7	0.011	0.228	0.913
	Rhizomicrobium	0.013	0.266	0.904
	Ruminiclostridium_9	0.011	0.225	0.915
	Saccharomyces	0.015	0.283	(0.899)
	Spirochaeta_2	0.000	0.022	0.984
	Staphylococcus	0.010	0.218	0.916
3-(methylthio)-1-Propanol	*[Eubacterium]_nodatum_group*	0.000	0.000	1.000
	Anaerofilum	0.005	0.139	0.940
	Cellulosimicrobium	0.015	0.290	(0.897)
	Escherichia-Shigella	0.041	0.520	(0.829)
	norank_c_Cyanobacteria	0.044	0.523	(0.823)
	norank_f_PHOS-HE51	0.005	0.139	0.940

	norank_o_Mollicutes_RF9	0.005	0.139	0.940
3,7-dimethyl-1,6-Octadien-3-ol	*[Eubacterium]_nodatum_group*	0.000	0.000	0.999
	Anaerofilum	0.007	0.176	0.929
	Cellulosimicrobium	0.013	0.266	(0.904)
	Escherichia-Shigella	0.044	0.523	(0.823)
	norank_c_Cyanobacteria	0.041	0.518	(0.831)
	norank_f_PHOS-HE51	0.007	0.176	0.929
	norank_o_Mollicutes_RF9	0.007	0.176	0.929
3-methyl-1-Butanol	*[Eubacterium]_eligens_group*	0.007	0.178	(0.928)
	Anaerotruncus	0.003	0.094	(0.954)
	Cloacibacterium	0.043	0.520	0.826
	Lachnospiraceae_NK4A136_group	0.027	0.423	(0.864)
	norank_f_Erysipelotrichaceae	0.025	0.404	(0.868)
	Odoribacter	0.031	0.456	(0.853)
4-methyl-1-Heptanol	*[Eubacterium]_nodatum_group*	0.005	0.128	0.944
	Anaerofilum	0.000	0.000	1.000
	Escherichia-Shigella	0.036	0.478	(0.841)
	norank_f_PHOS-HE51	0.000	0.000	1.000
	norank_o_Mollicutes_RF9	0.000	0.000	1.000
5-methyl-2-(1-methylethenyl)-4-Hexen-1-ol	*[Eubacterium]_nodatum_group*	0.005	0.128	0.944
	Anaerofilum	0.000	0.000	1.000
	Escherichia-Shigella	0.036	0.478	(0.841)
	norank_f_PHOS-HE51	0.000	0.000	1.000
	norank_o_Mollicutes_RF9	0.000	0.000	1.000
6-Methyl-1-octanol	*[Eubacterium]_hallii_group*	0.000	0.000	1.000
	Abiotrophia	0.003	0.091	0.955

	Acidisoma	0.001	0.053	0.968
	Aggregatibacter	0.002	0.058	0.966
	Capnocytophaga	0.000	0.005	0.993
	Caulobacter	0.007	0.176	0.930
	Cetobacterium	0.011	0.229	0.913
	Eikenella	0.000	0.001	0.997
	Gemella	0.016	0.294	0.896
	Geobacillus	0.002	0.058	0.966
	Hydrogenophilus	0.000	0.013	0.988
	Ignatzschineria	0.000	0.000	1.000
	Microbacterium	0.032	0.472	(0.849)
	Moraxella	0.038	0.495	0.837
	Neisseria	0.002	0.075	0.960
	norank_f_I-10	0.015	0.290	0.897
	Porphyromonas	0.002	0.069	0.962
	Pseudolabrys	0.001	0.053	0.968
	Pseudonocardia	0.000	0.000	1.000
	Solirubrobacter	0.030	0.453	0.854
	Sorangium	0.007	0.167	0.933
	unclassified_f_Rikenellaceae	0.014	0.266	0.904
	unclassified_o_Rhizobiales	0.029	0.448	0.856
	Undibacterium	0.003	0.080	0.959
	Veillonella	0.027	0.431	0.861
	Vibrionimonas	0.023	0.386	0.873
9,12-Octadecadien-1-ol	*[Eubacterium]_nodatum_group*	0.000	0.001	0.998
	Anaerofilum	0.009	0.203	0.922

	Cellulosimicrobium	0.013	0.251	(0.907)
	Enhydrobacter	0.048	0.558	(0.814)
	Escherichia-Shigella	0.046	0.539	(0.819)
	norank_c_Cyanobacteria	0.039	0.504	(0.835)
	norank_f_PHOS-HE51	0.009	0.203	0.922
	norank_o_Mollicutes_RF9	0.009	0.203	0.922
Octaethylene glycol	*[Eubacterium]_coprostanoligenes_group*	0.001	0.047	0.971
	Acinetobacter	0.018	0.318	0.890
	Alistipes	0.000	0.001	0.998
	Alloprevotella	0.000	0.017	0.986
	Anaerobiospirillum	0.000	0.004	0.994
	Anaerostipes	0.007	0.176	0.929
	Aspergillus	0.000	0.013	0.988
	Bacillus	0.005	0.128	0.944
	Blautia	0.015	0.288	0.898
	Burkholderia-Paraburkholderia	0.039	0.504	0.835
	Candidatus_Saccharimonas	0.000	0.004	0.994
	Catenibacterium	0.000	0.006	0.992
	Christensenellaceae_R-7_group	0.010	0.222	0.915
	Chroococcidiopsis	0.021	0.354	0.880
	Clostridium_sensu_stricto_15	0.000	0.015	0.987
	Coprococcus_3	0.002	0.065	0.964
	Desulfovibrio	0.000	0.001	0.997
	Devosia	0.000	0.004	0.995
	Dialister	0.002	0.072	0.961
	Dokdonella	0.034	0.474	0.846

Dyella	0.020	0.344	0.883
Enorma	0.000	0.004	0.994
Enterococcus	0.000	0.007	0.992
Erysipelotrichaceae_UCG-002	0.024	0.392	0.871
Faecalibacterium	0.000	0.009	0.990
Faecalibaculum	0.041	0.518	0.830
Family_XIII_AD3011_group	0.002	0.072	0.961
Haliangium	0.000	0.004	0.994
Helicobacter	0.001	0.040	0.973
Holdemanella	0.001	0.044	0.972
Inquilinus	0.000	0.004	0.994
Kroppenstedtia	0.012	0.249	0.908
Lachnospiraceae_UCG-010	0.000	0.004	0.994
Lawsonella	0.003	0.102	0.952
Limnohabitans	0.000	0.004	0.994
Marinobacter	0.000	0.004	0.994
Marvinbryantia	0.022	0.373	0.876
Megasphaera	0.019	0.337	0.885
Microlunatus	0.016	0.293	0.896
Mycobacterium	0.020	0.344	0.882
Nakamurella	0.002	0.070	0.962
Nitrolancea	0.001	0.039	0.974
Nitrospira	0.017	0.308	0.892
norank_c_Acidobacteria	0.004	0.119	0.947
norank_c_Bacteroidetes_BD2-2	0.000	0.004	0.994
norank_c_Bacteroidetes_VC2.1_Bac22	0.000	0.004	0.994

norank_c_BD7-11	0.001	0.044	0.972
norank_f_Acidimicrobiaceae	0.000	0.004	0.994
norank_f_Acidobacteriaceae_Subgroup_1	0.000	0.004	0.994
norank_f_Actinopolysporaceae	0.034	0.474	0.844
norank_f_B79	0.005	0.140	0.940
norank_f_Bacteroidales_BS11_gut_group	0.009	0.207	0.921
norank_f_Bacteroidales_S24-7_group	0.002	0.056	0.967
norank_f_Bacteroidales_UCG-001	0.030	0.452	0.855
norank_f_Chitinophagaceae	0.000	0.012	0.989
norank_f_Clostridiales_vadinBB60_group	0.000	0.004	0.994
norank_f_Gemmatimonadaceae	0.000	0.004	0.994
norank_f_JTB255_marine_benthic_group	0.000	0.004	0.994
norank_f_Lachnospiraceae	0.021	0.356	0.880
norank_f_MNG7	0.027	0.431	0.862
norank_f_Nitrosomonadaceae	0.000	0.025	0.982
norank_f_Rhodospirillaceae	0.002	0.058	0.966
norank_f_Xanthomonadaceae	0.000	0.007	0.991
norank_f_XIII	0.000	0.004	0.994
norank_o_AKYG1722	0.000	0.004	0.994
norank_o_Gaiellales	0.035	0.474	0.842
norank_o_Limnochordales	0.000	0.004	0.994
norank_o_Obscuribacterales	0.001	0.025	0.980
norank_o_Subgroup_7	0.000	0.004	0.994
norank_o_TRA3-20	0.015	0.282	0.899
norank_o_Xanthomonadales	0.000	0.025	0.982
norank_p_Parcubacteria	0.019	0.332	0.886

*norank_p_TM6_Dependentiae_*	0.000	0.004	0.994
Oceanobacillus	0.012	0.246	0.909
Odoribacter	0.006	0.164	0.934
Oribacterium	0.001	0.040	0.973
Oscillibacter	0.005	0.128	0.944
Paenibacillus	0.040	0.512	0.832
Papillibacter	0.000	0.004	0.994
Parabacteroides	0.000	0.011	0.989
Pelomonas	0.034	0.474	0.845
Perlucidibaca	0.000	0.004	0.994
Phascolarctobacterium	0.022	0.372	0.876
Prevotella_1	0.000	0.015	0.987
Prevotella_9	0.004	0.113	0.949
Prevotellaceae_NK3B31_group	0.000	0.025	0.982
Prevotellaceae_UCG-001	0.001	0.034	0.976
Prevotellaceae_UCG-003	0.000	0.000	1.000
Pseudoclavibacter	0.008	0.178	0.928
Pyramidobacter	0.000	0.004	0.994
Ralstonia	0.007	0.169	0.932
Rikenellaceae_RC9_gut_group	0.002	0.059	0.966
Rubellimicrobium	0.032	0.472	0.849
Ruminiclostridium	0.046	0.538	0.819
Ruminococcaceae_UCG-002	0.005	0.131	0.942
Ruminococcaceae_UCG-007	0.000	0.004	0.994
Ruminococcaceae_UCG-010	0.000	0.004	0.994
Ruminococcaceae_UCG-011	0.011	0.226	0.914

	Ruminococcus_1	0.000	0.005	0.993
	Ruminococcus_2	0.003	0.086	0.957
	Saccharomyces	0.014	0.273	(0.902)
	Saccharopolyspora	0.004	0.118	0.947
	Sedimentibacter	0.000	0.004	0.994
	Selenomonas	0.000	0.003	0.996
	Stenotrophomonas	0.018	0.318	0.890
	Streptococcus	0.027	0.424	0.863
	Streptomyces	0.043	0.520	0.826
	Subdoligranulum	0.000	0.005	0.993
	Succinivibrio	0.000	0.006	0.993
	Thalassobacillus	0.000	0.004	0.994
	Thermomonas	0.046	0.540	0.818
	Treponema_2	0.017	0.310	0.892
	Tyzzerella_3	0.000	0.004	0.994
	unclassified_f_Comamonadaceae	0.027	0.423	0.864
	unclassified_f_Coriobacteriaceae	0.000	0.004	0.994
	unclassified_f_Microbacteriaceae	0.000	0.004	0.994
	unclassified_f_Nocardioidaceae	0.000	0.004	0.994
	unclassified_f_Prevotellaceae	0.000	0.001	0.998
	unclassified_f_Xanthobacteraceae	0.037	0.490	0.838
	unclassified_f_Xanthomonadaceae	0.000	0.004	0.994
	unclassified_o_Chlamydiales	0.000	0.004	0.994
	unclassified_o_Solirubrobacterales	0.000	0.004	0.994
Phenylethyl Alcohol	*Bifidobacterium*	0.039	0.510	0.833
	Bradyrhizobium	0.014	0.271	0.902

Denitratisoma	0.012	0.249	0.908
Enhydrobacter	0.017	0.314	0.891
Lysinibacillus	0.028	0.434	0.860
norank_c_Cyanobacteria	0.027	0.423	0.864
norank_f_0319-6G20	0.047	0.543	0.818
norank_f_mle1-27	0.028	0.431	0.861
norank_p_SBR1093	0.021	0.359	(0.879)
Spirochaeta_2	0.047	0.547	0.817
Thauera	0.034	0.474	0.846
Thermoascus	0.012	0.243	0.910

Table S5. Significant microbiota for HA-producing.

Microbiota	quantiles	quantiles	significance	significance
Lactobacillus	0.1298	-0.0145	upper quantiles	No significance
Weissella	0.1105	0.0269	upper quantiles	No significance
norank_p__SBR1093	0.1113	0.0416	upper quantiles	No significance
Thermoascus	-0.1293	-0.0175	lower quantiles	No significance
Podosphaera	-0.1318	0.0214	lower quantiles	No significance
Paludibaculum	-0.1179	-0.0189	lower quantiles	No significance
norank_o__Acidimicrobiales	-0.1248	0.0192	lower quantiles	No significance
Bifidobacterium	-0.1306	-0.0019	lower quantiles	No significance
Scardovia	-0.1234	0.0161	lower quantiles	No significance
Acidothermus	-0.1234	0.0161	lower quantiles	No significance
Brevibacterium	-0.1217	0.0222	lower quantiles	No significance
Rothia	-0.1212	-0.0018	lower quantiles	No significance
Propioniciclava	-0.1234	0.0161	lower quantiles	No significance
norank_f__NS9_marine_group	-0.1234	0.0161	lower quantiles	No significance
Sphingobacterium	-0.1234	0.0161	lower quantiles	No significance
norank_f__Anaerolineaceae	-0.1326	0.0182	lower quantiles	No significance
norank_c__SJA-15	-0.1315	0.0208	lower quantiles	No significance
Staphylococcus	-0.1175	0.0454	lower quantiles	No significance
Anaerococcus	-0.1197	0.014	lower quantiles	No significance
Finegoldia	-0.1133	0.0479	lower quantiles	No significance
Lachnoanaerobaculum	-0.1093	0.0412	lower quantiles	No significance
Filifactor	-0.1348	-0.0091	lower quantiles	No significance
Schwartzia	-0.1234	0.0161	lower quantiles	No significance
Candidatus_Kuenenia	-0.1272	0.0178	lower quantiles	No significance

Woodsholea	-0.1234	0.0161	lower quantiles	No significance
norank_f__Holosporaceae	-0.1287	0.031	lower quantiles	No significance
Sandaracinobacter	-0.1271	0.0177	lower quantiles	No significance
Denitratisoma	-0.1089	-0.0161	lower quantiles	No significance
Sulfuritalea	-0.1297	-0.0076	lower quantiles	No significance
Thauera	-0.1334	0.0328	lower quantiles	No significance
norank_o__SC-I-84	-0.1234	0.0161	lower quantiles	No significance
Bdellovibrio	-0.1234	0.0161	lower quantiles	No significance
OM27_clade	-0.124	0.0123	lower quantiles	No significance
norank_f__BIrii41	-0.1264	0.0174	lower quantiles	No significance
Sulfuricurvum	-0.1202	0.0118	lower quantiles	No significance
Simiduia	-0.1234	0.0161	lower quantiles	No significance
Enhydrobacter	-0.1061	0.065	lower quantiles	No significance
norank_o__Run-SP154	-0.1234	0.0161	lower quantiles	No significance
norank_p__Saccharibacteria	-0.1174	-0.0211	lower quantiles	No significance
Pseudonocardia	0.0531	0.1107	No significance	upper quantiles
Solirubrobacter	0.0459	0.1135	No significance	upper quantiles
Porphyromonas	0.0639	0.1087	No significance	upper quantiles
Capnocytophaga	0.0519	0.1131	No significance	upper quantiles
norank_f__Simkaniaceae	-0.0678	0.1058	No significance	upper quantiles
Gemella	0.0138	0.1181	No significance	upper quantiles
[Eubacterium]_hallii_group	0.0531	0.1107	No significance	upper quantiles
Veillonella	-0.0116	0.109	No significance	upper quantiles
Ochrobactrum	0.0382	0.1078	No significance	upper quantiles
Methylobacterium	-0.0454	0.12	No significance	upper quantiles
unclassified_o__Rhizobiales	0.045	0.1115	No significance	upper quantiles

Achromobacter	-0.0444	0.1082	No significance	upper quantiles
Aquabacterium	-0.0168	0.1068	No significance	upper quantiles
Undibacterium	0.048	0.1169	No significance	upper quantiles
Eikenella	0.0572	0.1094	No significance	upper quantiles
Neisseria	0.0276	0.1092	No significance	upper quantiles
Aggregatibacter	0.0347	0.1144	No significance	upper quantiles
Haemophilus	-0.0604	0.1125	No significance	upper quantiles
Moraxella	0.0129	0.1244	No significance	upper quantiles
Ignatzschineria	0.0531	0.1107	No significance	upper quantiles

Table S6. Correlation between HA and significant microbiota on genus level.

Microbiota	HA	P value	P adjust	correlation
Pseudonocardia	6-Methyl-1-octanol	0	0	1
[Eubacterium]_hallii_group	6-Methyl-1-octanol	0	0	1
Ignatzschineria	6-Methyl-1-octanol	0	0	1
norank_f__NS9_marine_group	2-methyl-1-Propanol	2.26E-07	2.19E-05	0.999612
Woodsholea	2-methyl-1-Propanol	2.26E-07	2.19E-05	0.999612
Scardovia	2-methyl-1-Propanol	2.26E-07	2.19E-05	0.999612
Schwartzia	2-methyl-1-Propanol	2.26E-07	2.19E-05	0.999612
norank_o__SC-I-84	2-methyl-1-Propanol	2.26E-07	2.19E-05	0.999612
Bdellovibrio	2-methyl-1-Propanol	2.26E-07	2.19E-05	0.999612
Simiduia	2-methyl-1-Propanol	2.26E-07	2.19E-05	0.999612
Acidothermus	2-methyl-1-Propanol	2.26E-07	2.19E-05	0.999612
Propioniciclava	2-methyl-1-Propanol	2.26E-07	2.19E-05	0.999612
Sphingobacterium	2-methyl-1-Propanol	2.26E-07	2.19E-05	0.999612
norank_o__Run-SP154	2-methyl-1-Propanol	2.26E-07	2.19E-05	0.999612
Brevibacterium	2-methyl-1-Propanol	2.70E-06	0.000244	0.998658
Eikenella	6-Methyl-1-octanol	1.27E-05	0.001081	0.997083
norank_f__BIrii41	2-methyl-1-Propanol	2.17E-05	0.001734	0.996192
norank_o__Acidimicrobiales	2-methyl-1-Propanol	2.59E-05	0.001954	0.995841
Sulfuricurvum	2-methyl-1-Propanol	4.37E-05	0.003122	0.994596
Sandaracinobacter	2-methyl-1-Propanol	4.76E-05	0.003229	0.994362
Candidatus_Kuenenia	2-methyl-1-Propanol	5.57E-05	0.003596	0.993903
Capnocytophaga	6-Methyl-1-octanol	7.74E-05	0.004776	0.992807
OM27_clade	2-methyl-1-Propanol	0.000361	0.020736	0.984437
Anaerococcus	2-methyl-1-Propanol	0.000367	0.020736	0.984323

Rothia	2-methyl-1-Propanol	0.000611	0.033159	0.979751
Lachnoanaerobaculum	2-methyl-1-Propanol	0.001597	0.083338	0.967193
Aggregatibacter	6-Methyl-1-octanol	0.001692	0.085053	0.966221
Neisseria	1-Hexacosanol	0.00207	0.098606	0.962621
Porphyromonas	6-Methyl-1-octanol	0.002107	0.098606	0.962281
Neisseria	6-Methyl-1-octanol	0.002371	0.107258	0.959973
Undibacterium	6-Methyl-1-octanol	0.002531	0.110784	0.958638
norank_c__SJA-15	2-methyl-1-Propanol	0.003044	0.129075	0.954609
Ignatzschineria	1-Hexacosanol	0.003918	0.151908	0.948447
[Eubacterium]_hallii_group	1-Hexacosanol	0.003918	0.151908	0.948447
Pseudonocardia	1-Hexacosanol	0.003918	0.151908	0.948447
Finegoldia	2-methyl-1-Propanol	0.004563	0.164346	0.944326
Sulfuritalea	2-methyl-1-Propanol	0.004809	0.164346	0.942831
Ignatzschineria	1-Triacontanol	0.004889	0.164346	0.942351
Pseudonocardia	1-Triacontanol	0.004889	0.164346	0.942351
[Eubacterium]_hallii_group	1-Triacontanol	0.004889	0.164346	0.942351
Neisseria	1-Triacontanol	0.004966	0.164346	0.941899
Podosphaera	2-methyl-1-Propanol	0.00542	0.175127	0.939269
Veillonella	1-Hexacosanol	0.005798	0.182975	0.937166
Eikenella	1-Hexacosanol	0.006064	0.18703	0.935724
norank_f__Anaerolineaceae	2-methyl-1-Propanol	0.006285	0.189521	0.934553
Eikenella	1-Triacontanol	0.00699	0.206191	0.930939
Capnocytophaga	1-Hexacosanol	0.008917	0.257452	0.921875
Enhydrobacter	2-ethyl-1-Hexanol	0.009314	0.260578	-0.92013
norank_p__Saccharibacteria	2-(2-butoxyethoxy)-Ethanol	0.009409	0.260578	0.919717
Veillonella	1-Triacontanol	0.009986	0.271025	0.917258

Staphylococcus	2-phenoxy-Ethanol	0.010218	0.271883	0.916288
Capnocytophaga	1-Triacontanol	0.010806	0.281993	0.913879
Thermoascus	Phenylethyl Alcohol	0.011879	0.304154	0.909637
Denitratisoma	Phenylethyl Alcohol	0.012361	0.306436	0.907794
Aggregatibacter	1-Hexacosanol	0.01242	0.306436	0.90757
Enhydrobacter	1-Heptadecanol	0.013067	0.316637	-0.90515
Gemella	6-Methyl-1-octanol	0.015769	0.375401	0.895639
Denitratisoma	2-phenoxy-Ethanol	0.016624	0.388947	0.892792
Enhydrobacter	Phenylethyl Alcohol	0.017204	0.395695	0.890903
Lactobacillus	2-methyl-1-Propanol	0.01755	0.39692	-0.88979
Finegoldia	2-(2-butoxyethoxy)-Ethanol	0.019228	0.427747	0.884536
norank_p__SBR1093	Phenylethyl Alcohol	0.021135	0.462582	-0.87883
Filifactor	2-methyl-1-Propanol	0.022541	0.480649	0.874773
Aggregatibacter	1-Triacontanol	0.022669	0.480649	0.87441
Gemella	1-Triacontanol	0.023329	0.487034	0.872554
Gemella	1-Hexacosanol	0.023927	0.491962	0.870892
Undibacterium	1-Triacontanol	0.024375	0.493686	0.869662
Undibacterium	1-Hexacosanol	0.025587	0.510608	0.866385
Bifidobacterium	2-phenoxy-Ethanol	0.026804	0.527139	0.863168
Veillonella	6-Methyl-1-octanol	0.027489	0.532901	0.861385
norank_f__Holosporaceae	2-methyl-1-Propanol	0.028542	0.54137	0.85869
Anaerococcus	2-(2-butoxyethoxy)-Ethanol	0.028929	0.54137	0.85771
unclassified_o__Rhizobiales	6-Methyl-1-octanol	0.029471	0.54137	0.856349
Paludibaculum	2-methyl-1-Propanol	0.029522	0.54137	0.856222
Solirubrobacter	6-Methyl-1-octanol	0.030375	0.543486	0.854106
Porphyromonas	1-Hexacosanol	0.030438	0.543486	0.85395

Porphyromonas	1-Triacontanol	0.033271	0.586354	0.847121
Thauera	Phenylethyl Alcohol	0.033844	0.588801	0.845774
Filifactor	2-(2-butoxyethoxy)-Ethanol	0.03523	0.60515	0.84256
Enhydrobacter	2-Isopropyl-5-methyl-1-heptanol	0.037256	0.631956	-0.83796
Moraxella	6-Methyl-1-octanol	0.037841	0.633953	0.83666
Bifidobacterium	Phenylethyl Alcohol	0.039383	0.651741	0.833265
Achromobacter	2-ethyl-1-Hexanol	0.042286	0.691356	-0.82704
Thauera	2-methyl-1-Propanol	0.046158	0.745667	0.819038
norank_p__Saccharibacteria	2-ethyl-2-methyl-Tridecanol	0.047204	0.753594	0.816931
Enhydrobacter	9,12-Octadecadien-1-ol	0.048431	0.764194	-0.81449
